# Reciprocal regulation between GCN2 (eIF2AK4) and PERK (eIF2AK3) through the JNK-FOXO3 axis to modulate cancer drug resistance and clonal survival

**DOI:** 10.1016/j.mce.2020.110932

**Published:** 2020-09-15

**Authors:** Glowi Alasiri, Yannasittha Jiramongkol, Sasanan Trakansuebkul, Hui-Ling Ke, Zimam Mahmud, Kitti Intuyod, Eric W.-F. Lam

**Affiliations:** a Department of Surgery and Cancer, Imperial College London, Hammersmith Hospital Campus, London, W12 0NN, UK; b Department of Parasitology, Faculty of Medicine, Khon Kaen University, Khon Kaen, 40002, Thailand; c Cholangiocarcinoma Research Institute, Khon Kaen University, Khon Kaen, 40002, Thailand

**Keywords:** Forkhead transcription factor, FOXO3, Breast cancer, Chemotherapy, JNK, AKT, PERK, protein kinase R-like endoplasmic reticulum kinase, eIF2AK, Eukaryotic translation initiation factor 2-alpha kinase, GCN2, general control nonderepressible 2, ER, endoplasmic reticulum, MEFs, mouse embryo fibroblasts, AKT, protein kinase B, JNK, c-Jun N-terminal kinase, FOXO3, Forkhead box O3, PI3K, phosphatidylinositol 3-kinase, AMPK, adenosine monophosphate-activated-activated protein kinase

## Abstract

Pharmaceutical inhibitors of the endoplasmic reticulum (ER)-stress modulator PERK (eIF2AK3) have demonstrated anticancer activities in combination therapies, but their effectiveness as a single agent is limited, suggesting the existence of possible compensatory cellular responses. To explore the potential mechanisms involved, we performed time-course drug treatment experiments on the parental MCF-7 and drug resistant MCF-7Epi^R^ and MCF-7Tax^R^ breast cancer cells and identified GCN2 (eIF2AK4) as a molecule that can potentially cooperate with PERK to regulate FOXO3 via JNK and AKT to modulate drug response. Consistently, GCN2 knockdown severely impaired the clonal survival of parental and resistant MCF-7 cells and sensitised them to epirubicin and paclitaxel treatment. Western blot, RT-qPCR and ChIP analyses also confirmed that GCN2 inactivation causes an induction of JNK and thereby FOXO3 activity, culminating in an increase in PERK activity and expression at the transcription level. Conversely, PERK-inactivation using GSK2606414-induces an induction in GCN2 expression and activity also associated with JNK. In agreement, we also showed that the *perk*^−/−^ MEFs, expressing elevated levels of P-JNK, JNK, GCN2 and reduced levels of P-AKT and P-FOXO3, have lower clonogenicity and are more sensitive to epirubicin compared to wild-type MEFs. Similarly, *gcn2*^−/−^ MEFs expressing augmented levels of P-JNK, JNK, P-PERK, PERK and lower levels of P-AKT and P-FOXO3 also had lower clonogenicity and were more sensitive to epirubicin and PERK-inhibition. In addition, JNK1/2 deletion in MEFs resulted in reduced levels of GCN2, FOXO3, PERK, P-PERK expression as well as FOXO3 activity and enhanced clonal survival and resistance to PERK-inhibition. Together these results demonstrate that GCN2 cooperates with PERK through the JNK-FOXO3 axis in a reciprocal negative feedback loop to mediate cancer chemotherapeutic drug response and clonal survival, advocating the potential of targeting GCN2 as a therapeutic strategy for treating cancer and for overcoming drug resistance.

## Highlights

• Compensatory mechanisms compromise PERK (eIF2AK3) inhibitor response.
• GCN2 (eIF2AK4) cooperates with PERK to modulate cancer drug response.
• GCN2 and PERK regulate FOXO3 via JNK and AKT in response to cancer drug.
• GCN2 regulates PERK and vice versa in a reciprocal negative feedback loop.
• Inhibiting GCN2 is a novel strategy for targeting cancer and drug resistance.

## 1 Introduction

A healthy dynamic equilibrium between protein synthesis and degradation is required by cells to maintain protein homoeostasis (proteostasis), which in turn is critical for sustaining cell viability and growth. Endoplasmic reticulum (ER) plays a central role in preserving proteostasis through the control of protein synthesis, folding, quality control and distribution. Deregulated proteostasis results in the accumulation of misfolded or unfolded proteins and causes ER stress. This in turn activates a signalling cascade called the Unfolded Protein Response (UPR), which triggers a set of transcriptional and translational events to restore protein homeostasis, promoting cell survival and adaptation, or to induce cell death when proteostasis cannot be efficiently restored (Sarvani et al., 2017). It has also been proposed that cancer cells can manipulate UPR to acquire competitiveness and survival advantages to drive cancer initiation and progression as well as drug resistance (Salaroglio et al., 2017). In concordance, targeted suppression of UPR has been shown to improve therapeutic outcomes of multidrug resistant cancer cells (Alasiri et al., 2019; Garg et al., 2015; Urra et al., 2016)}.

Eukaryotic translation initiation factor 2 alpha kinases (eIF-2α kinases or eIF2AKs) are a family of four distinct serine-threonine kinases, including HRI (Heme-Regulated Inhibitor; eIF2AK1), PKR (RNA-dependent Protein Kinase; eIF2AK2), PERK (PKR-like ER Kinase; eIF2AK3) and GCN2 (General Control Non-derepressible 2; eIF2AK4). These eIF2AKs are key mediators of metabolic stress responses in mammalian cells and get activated upon distinct forms of metabolic stress signals. These eIF2AKs phosphorylate the alpha-subunit of eIF2 (eIF2α) to induce global protein translation inhibition and control cell survival upon different cellular metabolic stresses. Deregulation of EIF2AKs has been linked to numerous pathological conditions, such as diabetes, anaemia and other metabolic disorders, renal disorders, neurodegenerative disorders and cancers (Baird and Wek, 2012; Donnelly et al., 2013; Proud, 2005).

The Forkhead box O3 (FOXO3) transcription factor plays a pivotal role in promoting cell cycle arrest, senescence and cell death, as well as mediating the cytotoxic functions of cancer therapeutics. FOXO3 functions downstream of the phosphatidylinositol 3-kinase-protein kinase B (PI3K-PKB/AKT) signalling pathway as a tumour suppressor, preventing uncontrolled cellular proliferation. For example, FOXO3 regulates the expression of negative cell proliferation regulators, such as p27^Kip1^ and BIM (Dijkers et al., 2000; Essafi et al., 2005; Lam et al., 2006; Wang et al., 2013) but represses the expression of oncogenes, including FOXM1 (Karadedou et al., 2012; McGovern et al., 2009; Wang et al., 2013) to cause cell proliferative arrest, senescence and cell death (Lam et al., 2013). Like other tumour suppressors, FOXO3 is frequently downregulated or inactivated in different cancers and particularly, in drug-resistant cells, usually by deregulated hyperactive PI3K-AKT signalling (Ho et al., 2008; Wang et al., 2013). Conversely, several upstream regulatory kinases, such as c-Jun N-terminal kinase (JNK) and AMP-activated protein kinase (AMPK) can also stimulate nuclear localization and transcriptional activity of FOXO3 by phosphorylating specific sites (Zhao et al., 2011). PERK has previously been shown to phosphorylate FOXO3 directly in an AKT-independent mechanism to promote FOXO3 activity (Alasiri et al., 2018; Zhang et al., 2013, 2015).

Anthracyclines, including epirubicin, and taxanes, such as paclitaxel, are the most active and widely used chemotherapeutic agents for treating breast cancer, but the development of resistance to these chemotherapeutic agents often limits their efficacy (Moreno-Aspitia and Perez, 2009). Our previous work has shown that PERK mediates the cytotoxic signals of both the genotoxic and non-genotoxic chemotherapeutic drugs, including epirubicin and paclitaxel and has a role in modulating cancer drug resistance (Alasiri et al., 2019). We have also found that FOXO3 regulates PERK expression and that PERK expression is attenuated as a result of the adaptative lower FOXO3 expression in the drug-resistant breast cancer cells. Although the PERK small-molecule inhibitors, such as GSK2656157 and GSK2606414, have demonstrated good anti-tumour effects in mouse xenograft models (Atkins et al., 2013; Axten et al., 2012) and in combination treatments (Alasiri et al., 2019), their broader use as an anti-tumour drug has been hampered by their low efficacy as a single cytotoxic agent (Alasiri et al., 2019). This suggests the possible existence of parallel and/or compensatory survival pathways. An understanding of the molecular mechanism involved in PERK inhibitor insensitivity may suggest strategies for effective combination therapies and help to predict potential mechanisms of disease resistance to therapy targeting PERK.

Although different eIF2AKs recognise distinct cellular metabolic stress signals, a recent study has demonstrated that PERK (eIFA2K3) and (eIF2AK4) function cooperatively to promote cell survival during paclitaxel treatment *in vitro* and *in vivo* (Chen et al., 2019). Previous studies in Drosophila have also shown that both GCN2 and PERK can potentiate FOXO activity in response to ER stress (You et al., 2018,Zhang et al.). In agreement, a recent study also demonstrated that PERK and GCN2 activate MYC, a proto-oncogenic bHLH transcription factor which engages in tumour growth and proliferation, and its activation is responsible for the tumorigenesis of many human cancers (Tameire et al., 2019). Furthermore, PERK and GCN2 have been found to regulate Sesn2, an antioxidant protein involved in several stress conditions, which induce AMPK activity, autophagy and metabolic health (Jin et al., 2019). Collectively these previous findings led us to hypothesise that GCN2 may cooperate with PERK to modulate the cytotoxic drug response.

## 2 Materials and methods

### 2.1 Cell lines and culture

MCF-7, a human breast cancer cell line, was originally isolated in the American Type Culture Collection (Manassas, VA, USA) and was obtained from the Cell Culture Service, Cancer Research UK (London, UK), where it was tested and authenticated. The MCF-7Epi^R^ (resistant to epirubicin) and MCF-7Tax^R^ (resistant to paclitaxel) cell lines were generated in Prof. Eric Lam laboratory (Imperial College London, Hammersmith Hospital, UK) by culturing parental MCF-7 cells in increasing drug concentrations until they acquired resistance to 100 μmol/L of each drug (Khongkow et al., 2014, 2015). Mouse embryonic fibroblasts (MEFs) WT, *gcn2*^*−/−*^ and *perk*^*−/−*^ were originated from Dr. David Ron (University of Cambridge) and obtained from ATCC (LGC Standards – UK, Teddington Middlesex, UK) (Novoa et al., 2001). All cell lines were cultured in Dulbecco's Modified Eagle's Medium (Sigma-Aldrich, Irvine, UK) supplemented with 10% foetal calf serum (First Link Ltd, Birmingham, UK), 4 mM glutamine and 100 U/mL penicillin/streptomycin (Sigma-Aldrich) at 37 °C with 10% CO_2_. The drug resistant MCF-7Tax^R^ and MCF-7Epi^R^ cells were maintained with a supplement of 0.05 μM Paclitaxel (Hospira, Maidenhead, UK) and 17 μM epirubicin (Accord, Middlesex, UK), respectively. Adherent cultured cells were split when they reached 80% confluence. For sub-culturing, media was removed by an aspirator and the cells washed with room temperature PBS and then detached by adding 1 x Trypsin-EDTA. After incubation for 2 min at 37 °C, the trypsin was inactivated by adding complete media. Then the cells were counted using a haemocytometer and seeded in the suitable media volume into flask or plates.

#### 2.1.1 Western blot analysis

Western blotting was carried out on whole-cell extracts as previously described (Alasiri et al., 2019; Myatt et al., 2014) using the antibodies mentioned. FOXM1 (c-20; sc-502), P-PERK (Thr 981) (sc-32577), p27^Kip1^ (sc-528), Total JNK (SC-7345) and β-tubulin H-235; sc-9104) antibodies were purchased from Santa Cruz Biotechnology (CA, USA). The P-FOXO1 (Thr24)/FOXO3 (Thr32) (CST# 9464) and FOXO3 (CST#2497), PERK (CST#3192), P-AKT (S473) (CST#9271), P-AKT(T308) (CST#9275), AKT (CST#9272) GCN2 (CST#3302), P-JNK (CST#9251s) and eIF2α (CST#5324) were purchased from Cell Signaling Technology (New England Biolabs Ltd. Hitchin, UK). The P-elF2α antibody (ab32157; Abcam; Cambridge, UK). The primary antibodies (1:1000) were detected using horseradish peroxidase-conjugated secondary antibody (1:2000, DAKO, Glostrup, Denmark) and visualised using the ECL detection system (PerkinElmer Ltd, Beaconsfield, UK). Subcellular fractionation was performed as previously described (Myatt et al., 2014).

### 2.2 Gene silencing with small interfering RNAs (siRNAs)

To knockdown GCN2, ON-TARGET*plus* SMARTPool siRNAs (Dharmacon Thermo Scientific, CO, USA) were transfected into the cells using Oligofectamine (Invitrogen, UK) according to the manufacturer's instructions. The smart pool siRNAs were used to aim for all isoforms: GCN2 siRNA (L-005314-00-0005), and the NS control siRNA (D-001810-10-05). All the siRNAs were prepared by 1 X siRNA buffer (B-002000-UB-100) to acquire a final concentration of 20 μM, which was stored at −20 °C. After 48 h of transfection, cells were treated with epirubicin, GSK2606414 (VWR, Leicestershire, UK) or paclitaxel for the time points indicated and collected for analysis by western blot, RT-qPCR, SRB or clonogenic assays.

#### 2.2.1 Quantitative real time PCR (RT-qPCR)

RT-qPCR analysis was performed as described (Alasiri et al., 2019). Total RNA was extracted using the RNeasy Mini kit (Qiagen, Hilden, Germany). Complementary DNA was reverse-transcribed into cDNA using SuperScript Transcriptase III (Invitrogen) according to the manufacturer's protocol. Gene expressions were quantified via RT-qPCR, using Power SYBR Green PCR Master Mix (Applied Biosystems, Fisher Scientific UK Ltd, Loughborough, UK) and a standard curve as previously described (Kwok et al., 2008). L19, a housekeeping gene, was used as an internal control for normalization. Human primer sequences are L19-F 5′ GCGGAAGGGTACAGCCAAT3′, L19-R 5′ GCAGCCGGCGCAAA 3′, FOXO3-F 5′ TCTACGAGTGGATGGTGCGTT 3′, FOXO3-R 5′ CGACTATGCAGTGACAGGT3′, FOXM1-F 5′ TGCAGCTAGGGATGTGAATCTTC 3’, FOXM1-R 5′ GGAGCCCAGTCCATCAGAACT 3′, PERK-F 5′ TGGCCACTTTGAACTTCGGTA 3′, PERK-R 5′ CCACCCGGTTTAAAGGTGCT 3′, GCN2-F 5′ TGGATTTGAGGGTTAAATGCCC 3′, GCN2-R 5′ CCACAGTGTTTCTTGGCCAG 3′, p27^Kip1^-F5′CATTTGGTGGACCCAAAGAC3′,p27^Kip1^-R 5′CTTCTGAGGCCAGGCTTCTT3’. For mouse primer sequences are L19-F 5′GGTGCTTCCGATTCCAGAGT3′, L19-R 5′CCCATTCCCTGATCGCTTGA 3′, Foxo3-F 5′ CCGGACAAACGGCTCACT 3′, Foxo3-R 5′ GGCACACAGCGCACCAT 3′, Gcn2-F 5′ GGAAGAACTGGCCAAAAAGCA 3′, Gcn2-R 5′ TTCTCCTGAGCCTGCCTTTC 3′, Perk-F 5′GGATGTCGCCGATGGGATAG 3′, Perk-R 5′CGAAGTTCAAAGTGGCCAACA 3’.

#### 2.2.2 Chromatin immunoprecipitation (ChIP)

ChIP analysis was performed as described (Alasiri et al., 2019). The cell lines were transfected with ON-TARGET*plus* SMARTPool siRNAs GCN2 siRNA (L-005314-00-0005) and NS control siRNA (D-001810-10-05) (GE Dharmacon, Horizon Discovery LTD, UK), which are highly specific and have minimal off-target activity, were transfected into the cells using Oligofectamine (Invitrogen, Thermo Fisher Scientific, UK) according to the manufacturer's instructions. Then cells were then collected for the ChIP assay, as previously described. For immunoprecipitation, 2 μg of either IgG (P0447, DAKO) and FOXO3 (ab12162; Abcam) antibodies were added to the precleared samples. Then, a Wizard SV gel and PCR clean-up system quick protocol (Promega, Southampton, UK) was used to purify the DNA according to the manufacturer's instructions. For PCR reaction, 1 μL of DNA from each sample, 1 μL of mix of primers (50 nM final concentration), 5 μL SYBR green master mix (Applied Biosystems, Fisher Scientific UK Ltd, Loughborough, UK) and 3 μL DEPC-treated water per well were used. The reaction was run in 7900 HT Fast Real-time PCR System (Applied Biosystems) and the cycling program was 95 °C for 10 min followed by 40 cycles of 95 °C for 15 s, 60 °C for 30 s and 95 °C for 30 s, followed by a dissociation step. The pair of primers used for ChIP was: PERK pro-F 5′ GATGGCAGTGACCTGTGACA 3′ and PERK pro-R 5′ AGTCTTCTCCACTCTGCCCT3′. The control primers are Actin control-F 5′ AGCGCGGCTACAGCTTCA3′ and Actin control-R 5′ CGTAGCACAGCTTCTCCTTAATGT 3’. All experiments were done in triplicates and results were normalised to the IgG antibody.

#### 2.2.3 Clonogenic assay

Total of 3000 MCF-7, MCF-7-Epi^R^, MCF-7-Tax^R^ cells were seeded into six-well plates following GCN2 knockdown and left overnight for adherence, after which they were treated with increasing concentrations of GSK2606414 and has previously been described (Alasiri et al., 2019). Briefly after 48 h of incubation with the drug, cells were cultured in fresh drug-free media and grown for around 14 days until colony formation. Colonies were washed 3 times with PBS and fixed with 4% formaldehyde for 15 min at room temperature. After 3 additional washes with PBS, colonies were stained with 0.5% crystal violet (Sigma Aldrich) for 1 h, washed with flowing water, air-dried and quantified using ImageJ (https://imagej.nih.gov/).

#### 2.2.4 Statistical analysis

The data included in this paper are representative of 3 independent experiments, which were each completed in triplicate (presented as means ± SEM). GraphPad Prism was used for statistical analysis (version 5, San Diego, CA, USA), and two-tailed Student's t-test was used to compare the means. To compare between groups of more than two unpaired values, a one-way analysis of variance (ANOVA) was conducted. Two-way ANOVA was used between groups of 2 variables and was considered significant when *P < 0.05, **P < 0.01 and ***P < 0.001.

## 3 Results

### 3.1 GCN2 expression correlates negatively with FOXO3 and PERK expression after epirubicin and paclitaxel treatment

**Fig. 1:**
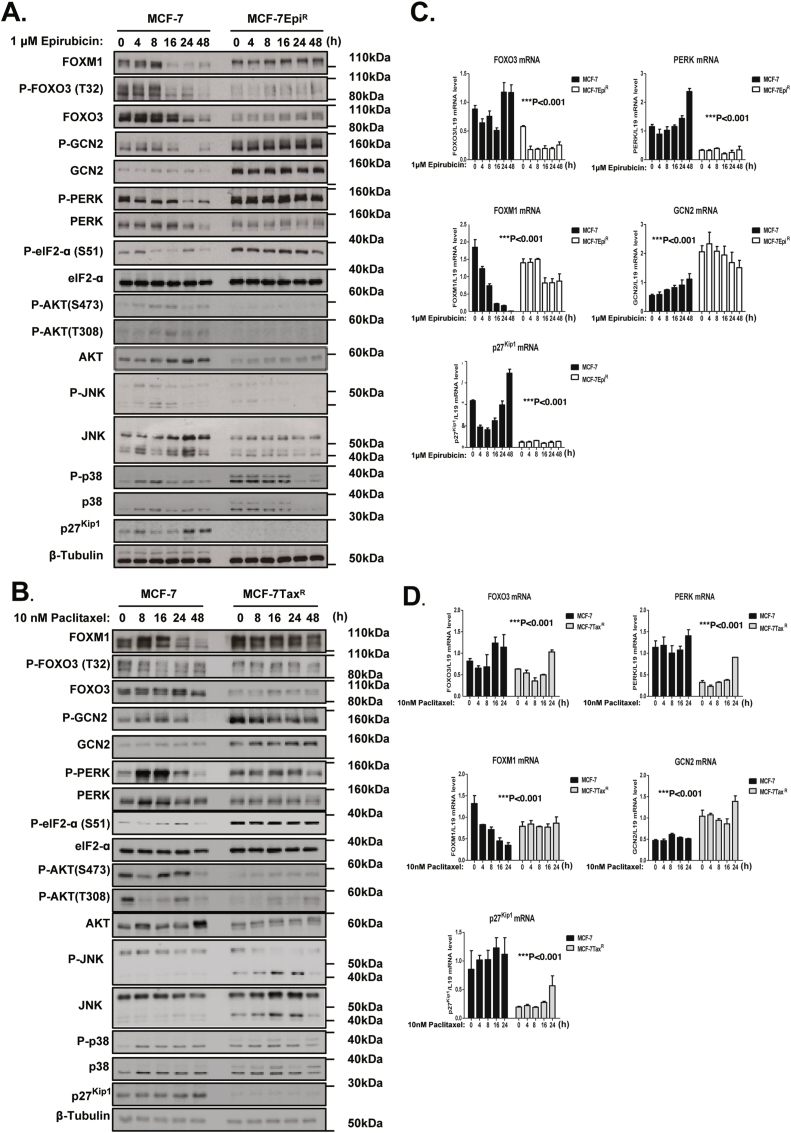
**Correlation between GCN2, PERK, JNK and FOXO3 expression and activity in response to epirubicin and palclitaxel treatment**. The epirubicin sensitive MCF-7 and resistant MCF-7Epi^R^ cells were either left untreated or treated with 1 μM epirubicin for the times shown. **B.** The paclitaxel sensitive MCF-7 and resistant MCF-7TaxR were either left untreated or treated with 10 nM paclitaxel for the times indicated. Whole-cell protein lysates were analysed by western blotting using the antibodies recognising the proteins indicated. Molecular weight markers are shown. The protein expression levels of P-FOXO3 (T32) (95 kDa), FOXO3 (95 kDa), FOXM1 (110 kDa) and ER stress molecules including P-GCN2 (160 kDa), GCN2 (220 kDa), P-PERK (140 kDa), PERK (140 kDa), P-eIF2a (38 kDa), eIF2a (38 kDa), p27^Kip1^ (27 kDa) and β-Tubulin (55 kDa) were investigated. **C.** Parallel RT-qPCRs were performed for studying GCN2, FOXO3, PERK, FOXM1, p27^Kip1^ mRNA expression following treatment with epirubicin. **D.** GCN2, FOXO3, PERK, FOXM1, p27^Kip1^ mRNA expression after treatment with paclitaxel was determined by RT-qPCR. Three technical repeats were performed in one experiment, and the data were normalised to L19 and showed as means ± S.E.M. The expression trends of mRNA species between MCF-7 and MCF-7Epi^R^ or c MCF-7Tax^R^ cells are compared using 2-way ANOVA (Significant ***p < 0.001, for all mRNA species, respectively). One of the three independent repeats is shown.

To test the conjecture that GCN2 compensates for PERK inactivation and to explore further the mechanism for PERK regulation, we investigated the effects of the chemotherapeutic drugs on PERK and GCN2 signalling in the drug sensitive and resistant MCF-7 breast cancer cells. Western blot analysis of the time-course experiments of epirubicin and paclitaxel treatments indicated that expression levels of FOXO3, PERK, p27^Kip1^ were higher in the MCF-7 compared with the drug-resistant MCF-7Epi^R^ and MCF-7Tax^R^ cells (Fig. 1A and C). Conversely, the levels of FOXM1 and GCN2 are constitutively higher in the drug resistant cells than the parental MCF-7 cells. In agreement, the GCN2 mRNA levels were lower in the parental MCF-7 compared with the MCF-7Epi^R^ and MCF-7Tax^R^ cells, while the mRNA levels of PERK, FOXO3 and p27^Kip1^ were higher in the MCF-7 cells compared with the drug resistant cells. (Fig. 1C and D). Importantly, P-GCN2 and P-PERK and their downstream effector P-eIF2α were augmented in MCF-7Epi^R^ and MCF-7Tax^R^ cells compared with the parental MCF-7 cells, suggesting that GCN2 and PERK cooperate to counteract the heightened ER stress in the drug resistant cells (Fig. 1A and B). Western blot analysis of the time-course experiments of epirubicin and paclitaxel treatments also indicated that both P-GCN2 and P-PERK were downregulated with similar kinetics in the MCF-7 cells but their expression levels remained constitutively high in the drug resistant cells, further suggesting that drug treatments repress both GCN2 and PERK activity in the sensitive but not in the resistant cells to mediate their cytotoxic effects. The results also revealed that the kinetics of JNK induction revealed by P-JNK occurred transiently before the downregulation of P-AKT and P-FOXO3 expression in response to drug treatment in the MCF-7 cells, confirming our previous findings that JNK mediates the effects of cytotoxic drugs through repressing AKT and thereby inducing FOXO3 activity (Sunters et al., 2006). This finding also suggested that JNK might function downstream of GCN2 and PERK to activate FOXO3, as both GCN2 and PERK have been shown to be the cellular targets of cytotoxic drugs (Alasiri et al., 2019). In contrast, the related total and phosphorylated forms of p38 MAPK were expressed at similar levels in both the parental and the drug resistant MCF-7 cells and their expression patterns were not strongly associated with GCN2, PERK and FOXO3 expression or activity. Together these data led us to hypothesise that both PERK and GCN2 cooperate to promote cytotoxic drug resistance and that cytotoxic drugs downregulated PERK and GCN2 activity to induce JNK and FOXO3 activation in the drug sensitive MCF-7 cells.

### 3.2 GCN2 knockdown depletes clonogenic capacity and increases sensitivity to cytotoxic agents

**Fig. 2:**
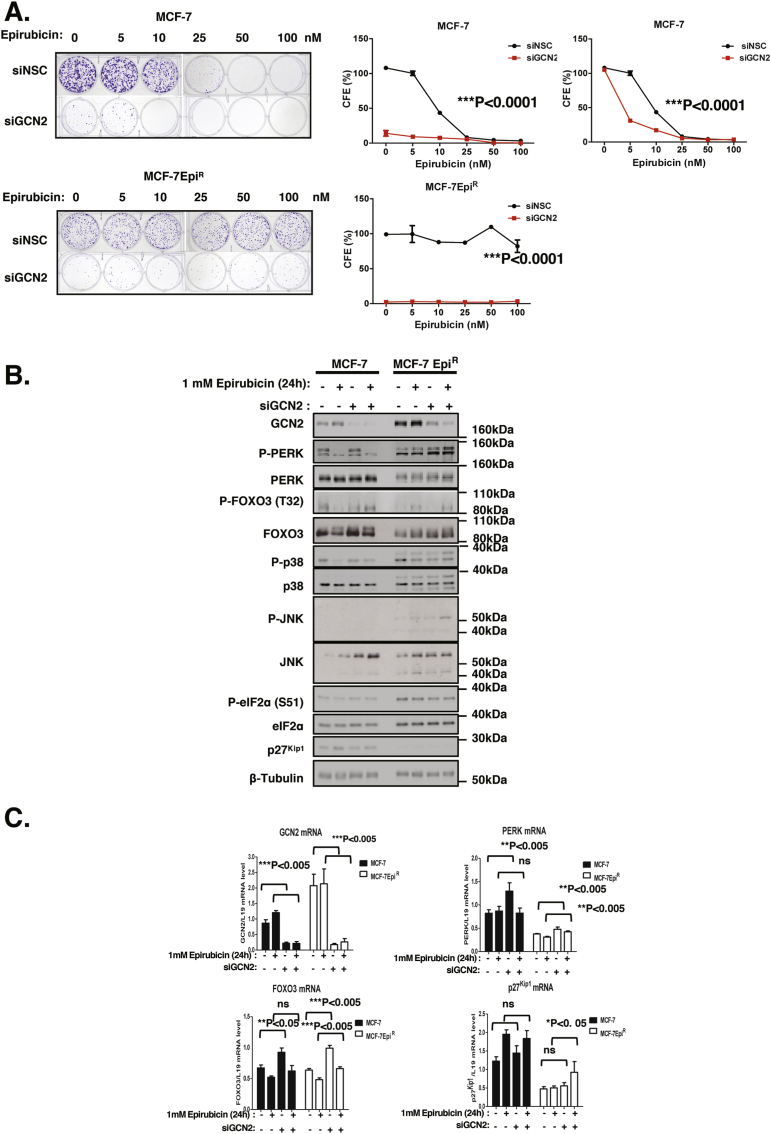
**Effects of silencing GCN2 on MCF-7 and MCF-7Epi**^**R**^**cells treated with epirubicin**. **A.** MCF-7 and MCF-7Epi^R^ cells were treated with 0, 5 nM, 10 nM, 25 nM, 50 nM and 100nM of epirubicin for 48 h. Fresh media were then added and maintained for 10 days before staining violet in clonogenic assays. The clonogenic results of the cells were normalised to that of the MCF-7 or MCF-7Epi^R^ cells without GCN2 knockdown and epirubicin treatment. The clonogenic results of the cells were normalised to that of MCF-7 or MCF-7Epi^R^ cells without epirubicin treatment (far right panel). Data are representative of 3 independent experiments. Data represent means ± SEM. Significant *P < 0.05, ***P < 0.001. (n = 3; 1-way ANOVA). Significant *P < 0.05, ***P < 0.001. **B.** Expression levels of signalling molecules were analysed by parallel Western blotting after transfection with siRNA in the presence of 1 μM epirubicin. Western blotting was performed to determine the protein expression levels for GCN2 (220 kDa), FOXO3 (85 kDa), P-PERK (140 kDa), PERK(140 kDa), P-eIF2a (38 kDa), eIF2a (38 kDa), P-p38 (38 kDa), p38 (38 kDa), P-JNK (54 kDa), JNK (54 kDa), p27^Kip1^ (27 kDa) and β-Tubulin (55 kDa). The ratio to tubulin expression calculated the relative P-PERK, PERK and FOXO3 expression levels. **C.** Expression levels of GCN2, FOXO3,PERK and p27^Kip1^ mRNA were investigated by RT-qPCR, and the data normalised with L19 RNA levels and displayed as means ± SEM (n = 3; 2 tailed *t*-test). Significant *P < 0.05, **P < 0.01. Representative RNA expression profiles of one of at least 3 independent experiments are shown.

We have previously demonstrated that PERK furthers cytotoxic drug resistance by repressing FOXO3 (Alasiri et al., 2019). To test the role GCN2 plays in cancer cell drug resistance, GCN2 was depleted using siRNA in both the parental MCF-7 and the drug resistant MCF-7Epi^R^ cells. GCN2 knockdown decreased the clonogenicity of MCF-7 cells substantially and sensitised them to epirubicin treatment (Fig. 2A). Interestingly, GCN2 silencing almost completely depleted the clonogenic capacity of the epirubcin resistant MCF-7Epi^R^ cells (Fig. 2A). Together, these results suggest that GCN2 has a key role in clonal survival and epirubicin resistance in these breast cancer cells. Parallel Western blot analysis showed effective GCN2 knockdown in both parental and epirubicin-resistant MCF-7 cells. The results also demonstrated that GCN2 knockdown caused an induction in JNK and PERK expression as well as their activity, as revealed by P-PERK and P-JNK expression, particularly in the MCF-7Epi^R^ cells (Fig. 2B and Supplementary Fig. S1). This JNK and PERK induction was associated with an increase in FOXO3 expression, suggesting a potential feedback compensatory mechanism involving the induction of JNK, FOXO3 and PERK expression and their activity. In agreement, RT-qPCR analysis showed that the mRNA levels for p27^Kip1^, PERK and FOXO3, all downstream targets of FOXO3, increased significantly in both sensitive and resistant MCF-7 cells following GCN2 silencing (Fig. 2C). Notably, although GCN2 depletion caused a downregulation in P-eIF2α expression in the MCF-7Epi^R^, it did not have any discernible effects on P-eIF2α expression in the parental MCF-7 cells, indicating further a compensatory mechanism exists to alleviate ER and other cytotoxic stresses.

**Fig. 3:**
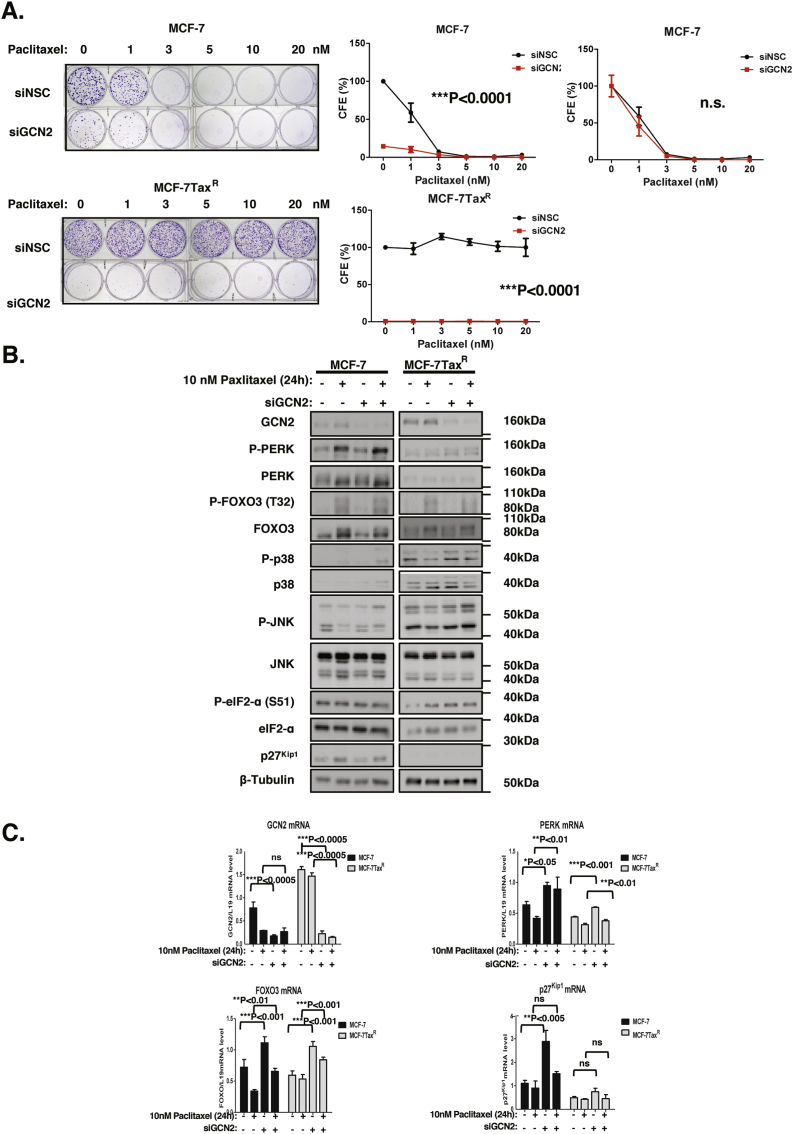
**Effects of silencing GCN2 on MCF-7 and MCF-7Tax**^**R**^**cells treated with paclitaxel**. **A.** MCF-7 and MCF-7Tax^R^ cells were treated with 0, 1 nM, 3 nM, 5 nM, 10 nM and 20 nM of paclitaxel for 48 h. Fresh media were then added and maintained for 10 days before staining violet in clonogenic assays (n = 3; 2-way ANOVA). The clonogenic results of the cells were normalised to that of the MCF-7 or MCF-7-Tax^R^ cells without GCN2 knockdown and paclitaxel treatment. The clonogenic results of the cells were also normalised to that of MCF-7 cells without paclitaxel treatment (far right panel). Data are representative of 3 independent experiments. Data represent means ± SEM. (n = 3; 2-way ANOVA). Significant *P < 0.05, ***P < 0.001. Significant, ***P < 0.001; ns, not significant. **B.** Expression levels of signalling molecules were analysed by parallel Western blotting after transfection with siRNA in the presence of 10 nM paclitaxel. Western blotting was performed to determine the protein expression levels for GCN2 (220 kDa), FOXO3 (85 kDa), P-PERK (140 kDa), PERK(140 kDa), P-eIF2a (38 kDa), eIF2a (38 kDa), P-p38 (38 kDa), p38 (38 kDa), P-JNK (54 kDa), JNK (54 kDa), p27^Kip1^ (27 kDa) and β-Tubulin (55 kDa). The ratio to tubulin expression calculated the relative P-PERK, PERK and FOXO3 expression levels. **C.** Expression levels of GCN2, FOXO3,PERK and p27^Kip1^ mRNA were investigated by RT-qPCR, and the data normalised with L19 RNA levels and displayed as means ± SEM (n = 3; 2 tailed *t*-test). Significant *P < 0.05, **P < 0.01. ***P < 0.001. Representative RNA expression profiles of at least 3 independent experiments are shown.

To investigate further the role of GCN2 in drug resistance and clonal survival, MCF-7 and the paclitaxel-resistant MCF-7Tax^R^ cells were subjects to GCN2 knockdown and paclitaxel treatment (Fig. 3). Consistent with the epirubicin treatment results, GCN2 knockdown severely impaired the clonal viability of MCF-7 cells and sensitised them to paclitaxel treatment (Fig. 3A). Similarly, GCN2 depletion completely obliterated the clonogenicity of the paclitaxel-resistant MCF-7Tax^R^ cells, further confirming that the drug-resistant cancer cells are heavily dependent on GCN2 for clonal survival. Together these results provided strong indication that GCN2 plays a pivotal role in cancer cell clonal renewal and survival as well as drug resistance. Western blot results again showed efficient GCN2 knockdown and revealed that GCN2 knockdown induced P-PERK expression in both the MCF-7 and MCF-7Tax^R^ cells (Fig. 3B and Supplementary Fig. S2). In concordance, the expression levels of P-JNK were increased evidently in MCF-7Tax^R^ and to a lesser extent in MCF-7 cells. In addition, FOXO3 expression levels were induced in both parental and paclitaxel-resistant MCF-7 cells following GCN2 depletion, while total PERK was induced only in MCF-7 but not in MCF-7Tax^R^ cells. The results also revealed that eIF2α activity shown by P-eIF2α did not reduce after GCN2 loss, which might be due to the compensatory induction of P-PERK. Consistently, RT-qPCR analysis showed that the mRNA levels of FOXO3 targets, PERK, FOXO3 and p27^Kip1^, were mostly augmented following GCN2 depletion, indicating an induction of FOXO3 activity in response to GCN2 loss (Fig. 3C). In addition, GCN2 also appears to be involved in a more predominant role than PERK in promoting drug resistance and clonal survival, as its depletion has more severe consequences on clonal viability. Together these data suggest a model where GCN2 and PERK cooperate to promote resistance to ER stress and cytotoxic drug action through repressing FOXO3 via activating AKT and restricting JNK activity.

### 3.3 GCN2 and PERK cooperate to modulate the effects of cytotoxic agents and their resistance

**Fig. 4:**
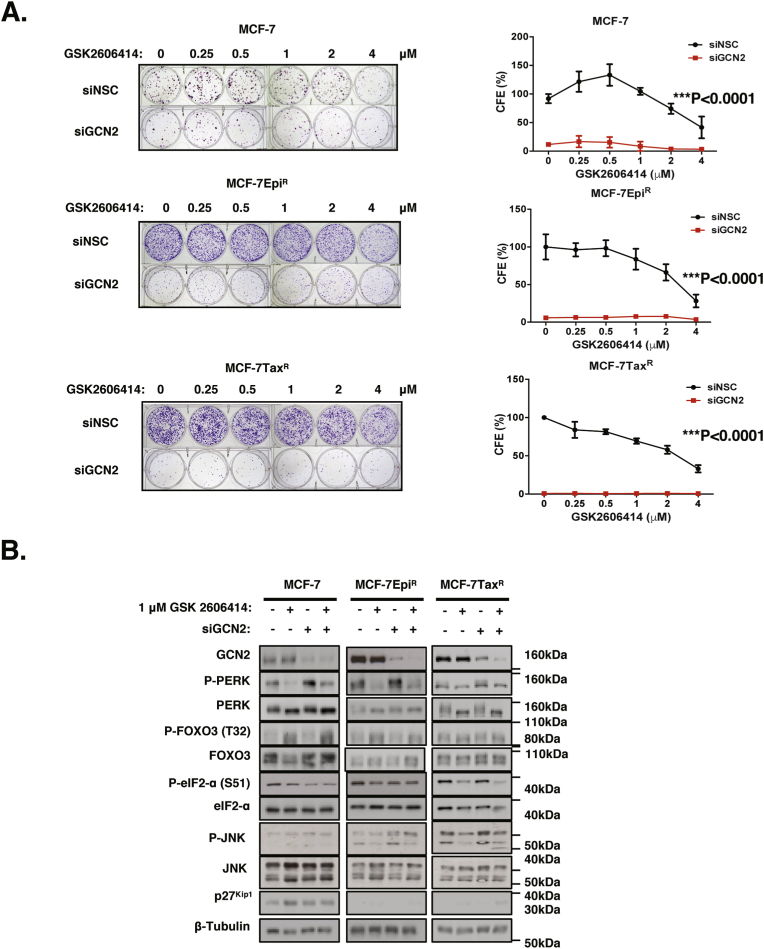
**Effects of silencing GCN2 on clonogenicity and protein expression in MCF-7, MCF-7-Epi**^**R**^**and MCF-7Tax**^**R**^**treated with GSK2606414**. MCF-7, MCF-7Epi^R^ and MCF-7Tax^R^ cells were transfected with either control or GCN2 siRNA and then treated with GSK2606414. **A.** MCF-7, MCF-7Epi^R^ and MCF-7Tax^R^ cells were treated with different concentrations of GSK2606414 every 48 h for 10 days after GCN2 depletion using siRNA. Fresh media were added and cells maintained for 10 days then stained with crystal violet in clonogenic assays. Representative images are shown. The clonogenic results of the cells were normalised to that of MCF-7, MCF-7-Epi^R^ or MCF-7-Tax^R^ cells without GCN2 silencing and GSK2606414 treatment (right panel). Data are representative of 3 independent experiments. Data represent means ± SEM. Significant *P < 0.05, ***P < 0.001. (n = 3; 2-way ANOVA). Significant *P < 0.05, ***P < 0.001. **B.** Expression levels of protein were then analysed after transfection with either control or GCN2 siRNA with and without 1 μM GSK2606414 treatment. Western blotting was performed to determine the protein expression levels for GCN2 (220 kDa), FOXO3 (85 kDa), P-PERK (140 kDa), PERK (140 kDa), P-eIF2a (38 kDa), eIF2a (38 kDa), P-p38 (38 kDa), p38 (38 kDa), P-JNK (54 kDa), JNK (54 kDa), p27^Kip1^ (27 kDa) and β-Tubulin (55 kDa).

**Fig. 5:**
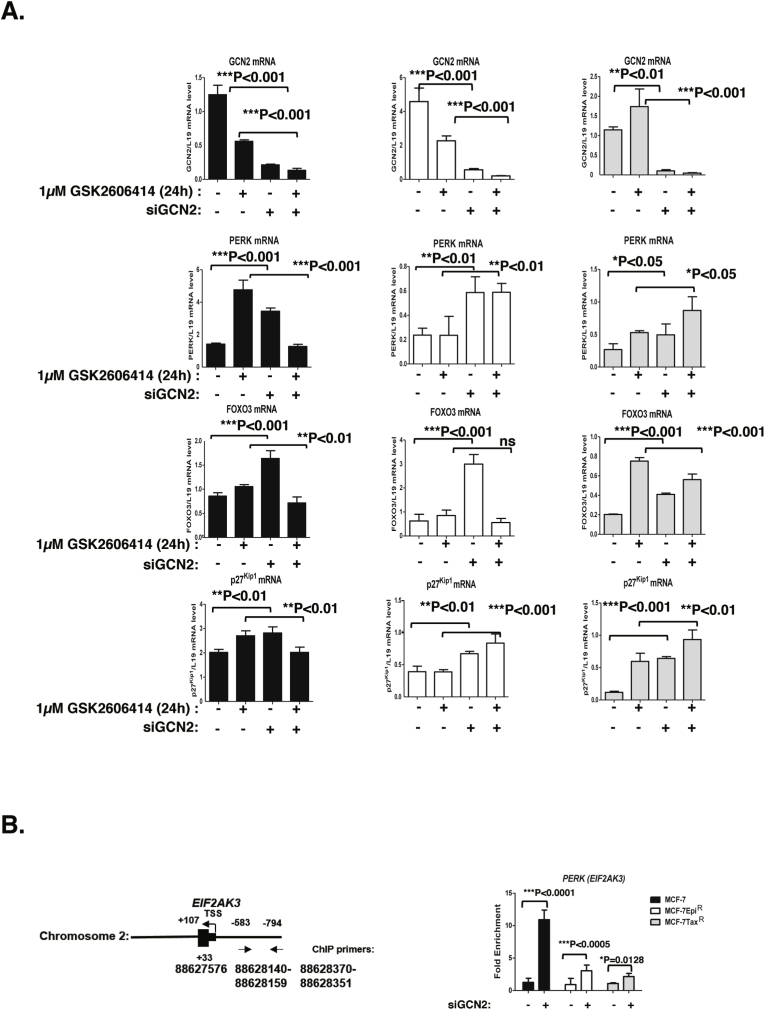
**Effects of silencing GCN2 on mRNA expression and FOXO3 recruitment to *PERK* in MCF-7, MCF-7-Epi**^**R**^**and MCF-7Tax**^**R**^**treated with GSK2606414**. MCF-7, MCF-7Epi^R^ and MCF-7Tax^R^ cells were treated with 1 μM of GSK2606414 for 48 h after GCN2 depletion using siRNA. **A.** GCN2, FOXO3, PERK and p27^Kip1^ mRNA levels were investigated using RT-qPCR and the data normalised with L19 RNA level and displayed as means ± SEM (n = 3; 2 tailed *t*-test). Significant *P < 0.05, **P < 0.01. Representative RNA expression profiles of at least 3 independent experiments are shown. **B.** FOXO3-binding site on human *PERK* promoter. ChIP-sequencing data of FOXO3-binding in DLD1 colon carcinoma cells (Eijkelenboom et al., 2013) were used for predicting FOXO3-binding sites on *PERK* gene using the Integrative Genomics Viewer (Version 2.3.88) and the hg19 UCSC Genome Browser 45 (left panel). ChIP analysis of FOXO3 binding on *PERK* promoter in MCF-7, MCF-7-EpiR cells and MCF-7TaxR transfected with non-trageting controls (siNSC) or siRNA targeting GCN2 (siGCN2). Representative ChIP analysis profiles of at least 3 independent experiments are shown (right panel). Three technical repeats were conducted in one experiment, and data were normalised to IgG and displayed as means ± SEM (n = 3; two-way ANOVA). Significant: *P < 0.05, **P < 0.01, ***P < 0.001.

To investigate further the mechanism by which GCN2 and PERK cooperate to confer tolerance to cytotoxic agents, we subjected the MCF-7, MCF-7Epi^R^ and MCF-7Tax^R^ cells to GCN2 siRNA knockdown and/or the PERK inhibition by GSK2606414 and investigated their response to GCN2 and PERK inactivation. In agreement with the previous data with epirubicin and paclitaxel treatments, clonogenic survival assays showed that GCN2 knockdown severely impaired the clonal survival of the parental MCF-7 cells and sensitised them to PERK inactivation (Fig. 4A). Similarly, GCN2 silencing almost completely depleted the clonal viability of the MCF-7Epi^R^ and MCF-7Tax^R^ drug resistant breast cancer cells (Fig. 4A). Western blot analysis demonstrated efficient GCN2 depletion in MCF-7, MCF-7Epi^R^ and MCF-7Tax^R^ cells after siRNA-mediated knockdown (Fig. 4B). In agreement, the results also demonstrated that GCN2 knockdown led to an induction of PERK protein expression in both MCF-7, MCF-7Epi^R^ but not MCF-7Tax^R^ cells. Critically, following GCN2 depletion, the induction of PERK expression in MCF-7 was substantially higher than in MCF-7Epi^R^ than MCF-7Tax^R^ cells. This probably is responsible for MCF-7 cells being relatively more refractory to GCN2 depletion in clonogenic assays compared to the drug-resistant cells, as PERK is induced more efficiently in MCF-7 compared with MCF-7Epi^R^ and MCF-7Tax^R^ cells to compensate for the loss of GCN2. The induction of PERK is ineffective in MCF-7Epi^R^ and MCF-7Tax^R^, possibly to be partly due to their low intrinsic FOXO3 expression and activity. These results also provided further evidence that the induction of PERK expression and thereby its activity are mediated by FOXO3 as previously published (Alasiri et al., 2019). The results further suggested that GCN2, like PERK, negatively regulates FOXO3 to promote clonal survival and drug resistance (Alasiri et al., 2019). In addition, JNK phosphorylation was similarly induced with its total protein levels in the parental MCF-7 and resistant MCF-7Epi^R^ and MCF-7Tax^R^ cells, suggesting JNK levels could be limiting in relation to upstream signals. Notably, FOXO3 protein levels were increased substantially in the parental MCF-7 and the drug resistant MCF-7Epi^R^, but only marginally in the MCF-7Tax^R^ cells. Next, the transcript mRNA levels were analysed using RT-qPCR. In agreement with the protein expression results, PERK and FOXO3 mRNA levels were elevated after GCN2 depletion (Fig. 5A). The p27^Kip1^ mRNA levels were also induced by GCN2 depletion, providing evidence for FOXO3 activation in MCF-7 cells following GCN2 depletion. Notably, GCN2 silencing plus PERK inhibition caused a significant reduction in PERK, p27^Kip1^, FOXO3 mRNA levels, which could be the consequence of high degrees of cell death. Nevertheless, together these results further demonstrate that GCN2 depletion causes an induction in FOXO3 activity and thereby PERK expression and its activity. GCN2 also crosstalks with PERK through the JNK-FOXO3 axis and that GCN2 is crucial for the survival of drug-resistant cells. Next, we considered if the regulation of PERK by FOXO3 in response to GCN2 depletion is mediated at the promoter level. Analysis of a ChIP-seq dataset from the DLD1 colon carcinoma cells (Eijkelenboom et al., 2013) confirmed that FOXO3 binds to the promoter region of *PERK* gene (Fig. 5B). ChIP analysis was then performed with primers designed to recognise a FOXO3-binding region −583 and −794 (bp) upstream of the *PERK* gene on the MCF-7, MCF-7-Epi^R^ and MCF-7Tax^R^ cells in the absence or presence of GCN2 knockdown (Fig. 5B). Enrichment of FOXO3 binding was detected in this upstream region of the *PERK* promoter region, indicating that FOXO3 can regulate PERK expression through its promoter level. The ChIP results also indicated that GCN2 depletion increased the recruitment of FOXO3 to the PERK promoter in all three MCF-7 cell lines, with the levels of induction much higher in the MCF-7 cells compared with the MCF-7-Epi^R^ and MCF-7Tax^R^ cells. Together these results suggest that PERK expression can be induced by GCN2 in a compensatory mechanism involving FOXO3. (Fig. 5B).

### 3.4 PERK deletion in MEFs induces JNK and FOXO3 activity and GCN2 expression

**Fig. 6:**
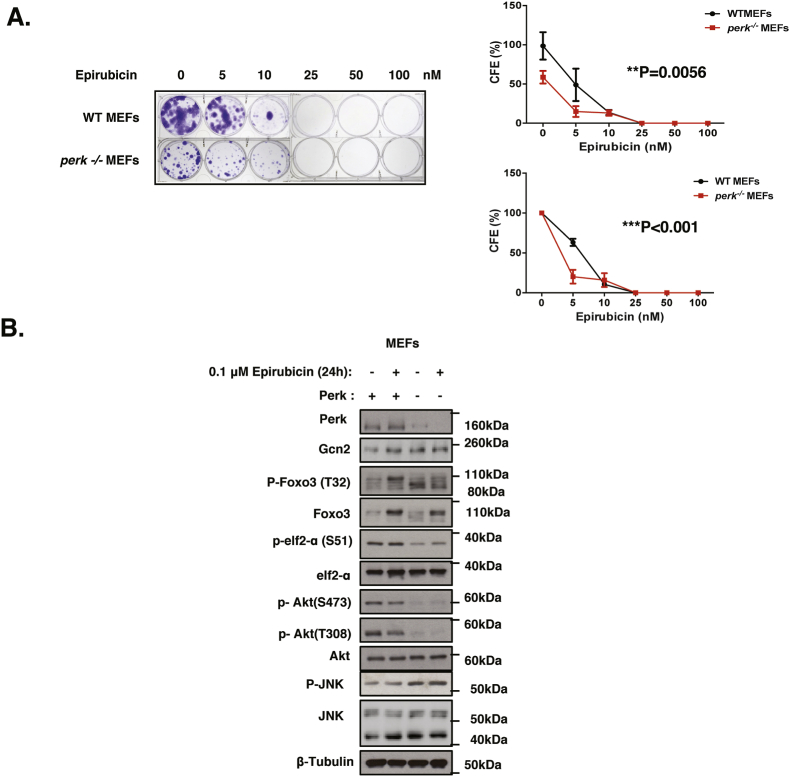
**Effects of PERK deletion on protein expression and clonogenicity of MEFs in response to epirubicin**. **A.** WT and *perk*-deficient MEFs were treated with 0, 5, 10, 25, 50 and 100 nM of epirubicin for 48 h, then incubated with fresh media for 10 days and stained with crystal violet in clonogenic assays. Representative images of at least 3 independent experiments are displayed. The clonogenic results of the cells were normalised to that of the WT MEFs without epirubicin treatment (Top right panel). The clonogenic results of the cells were also normalised to that of the respective WT and *perk*-deficient MEFs without epirubicin treatment (Bottom right panel) Data are representative of 3 independent experiments. Data represent means ± SEM. (n = 3; 2-way ANOVA). Significant *P < 0.05, ***P < 0.001. Significant, ***P < 0.001; ns, not significant. **B.** Expression levels of protein in WT and *perk*^*−/−*^ MEFs in response to 0.1 μM epirubicin (24 h). Western blot analysis was carried out fto determine the expression of P-Foxo3 (T32) (95 kDa), Foxo3 (85 kDa), Gcn2 (220 kDa), Perk (140 kDa), P-eIF2a (38 kDa), eIF2a (38 kDa), P-JNK (54 kDa), JNK (54 kDa), P-AKT (S473) (60 kDa), P-AKT (T308) (60 kDa), AKT (60 kDa) and β-Tubulin (55 kDa).

To investigate further the potential compensatory mechanism between GCN2 and PERK, western blot analysis and clonogenic assay were performed on *perk* knockout (*perk*^*−/−*^) and wild-type (WT) mouse embryonic fibroblasts (MEFs) in response to epirubicin and PERK inactivation by GSK2606414 (Fig. 6; Supplementary Fig. S3). Consistent with the GSK2606414 treatment results in MCF-7 cells, the clonogenic assay results showed that *perk*^*−/−*^ MEFs cells had a lower intrinsic clonal viability compared to WT MEFs and that *perk*^*−/−*^ MEFs were also more sensitive to epirubicin treatment (Fig. 6A; Supplementary Fig. S3). In addition, the expression levels of GCN2, P-JNK and FOXO3 were elevated in cells with PERK deletion or inhibition (Fig. 6B; Supplementary Figs. S3 and S4). These results further confirmed that GCN2 is upregulated to compensate functionally for PERK loss and suggested that JNK is involved in this crosstalk. Furthermore, the clonogenic assays on wild-type and PERK-deficient MEFs with GSK2606414 treatment also showed that the inhibitor is specific for PERK and it inhibits the clonogenicity of WT but has no effects on PERK-deficient MEFs at concentration ranges used in our experiments (Supplementary Fig. S3).

### 3.5 GCN2 deletion in MEFs induces JNK and FOXO3 activity and PERK expression

**Fig. 7:**
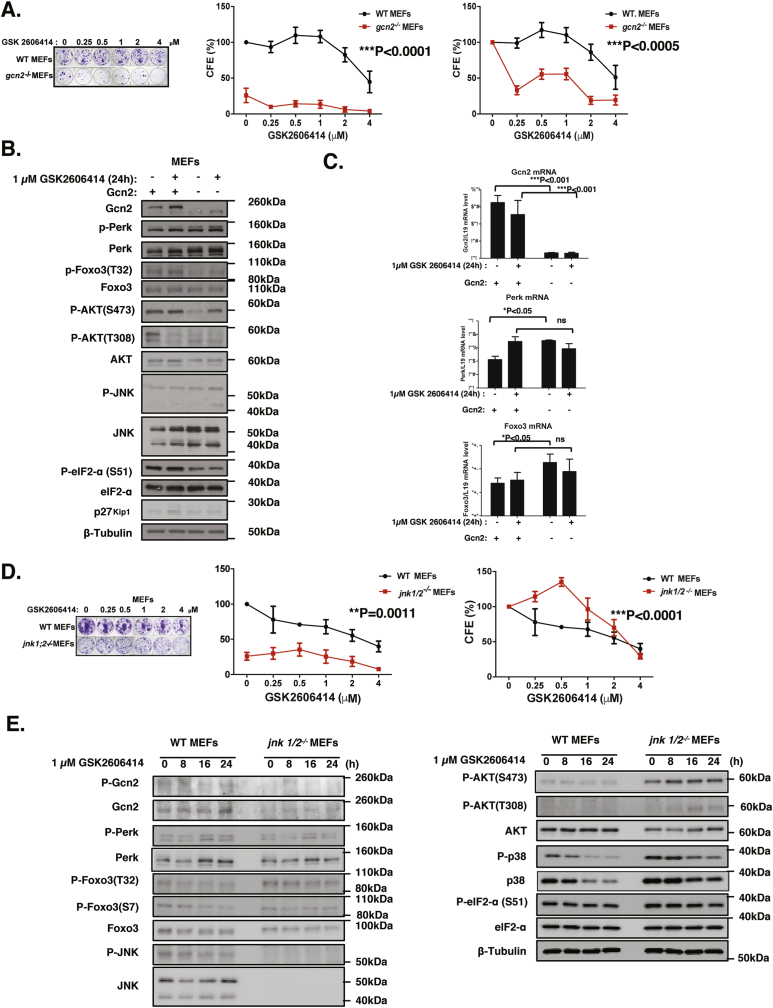
**GCN2 and JNK deletion in MEFs modulates JNK-FOXO3 activity and PERK/GCN2 expression**. **A.** WT and *gcn2*^*−/−*^ MEFs were treated and 0, 0.25, 0.5, 1, 2, 4 μM GSK2606414 every 48 h for 10 days. The resultant cells were stained with crystal violet in clonogenic assays. Representative images of at least 3 independent experiments are shown. The clonogenic results of the cells were normalised to that of the WT MEFs without epirubicin treatment (middle panel). The clonogenic results of the cells were also normalised to that of the respective WT and *gcn2*-deficient MEFs without GSK2606414 treatment (right panel) Data are representative of 3 independent experiments. Data represent means ± SEM. (n = 3; 2-way ANOVA). Significant *P < 0.05, ***P < 0.001. Significant, ***P < 0.001; ns, not significant. **B.** Expression levels of protein and mRNA for WT and *gcn2*^*−/−*^ MEFs in response to 1 μM GSK2606414. Expression for P-Foxo3 (T32) (95 kDa), Foxo3 (85 kDa), Gcn2 (220 kDa), Perk (140 kDa), P-eIF2a (38 kDa), eIF2a (38 kDa), P-JNK(54 kDa), JNK (54 kDa), P-AKT (S473) (60 kDa), P-AKT (T308) (60 kDa), AKT (60 kDa) and β-Tubulin (55 kDa). **C.** Foxo3, Gcn2, and Perk mRNA expression was determined by RT-qPCR (Two-Way ANOVA). **D.** WT MEFs and *jnk1/2*^*−/−*^ MEFs were treated with different concentrations of GSK2606414 for 48 h. Fresh media were then added and cells maintained for 10 days before staining with crystal violet in clonogenic assays. Representative images of at least 3 independent experiments are shown. The clonogenic results of the cells were normalised to that of the WT MEFs without epirubicin treatment (Top right panel). The clonogenic results of the cells were also normalised to that of the respective WT and *jnk1/2*-deficient MEFs without GSK2606414 treatment (Bottom right panel). Data are representative of 3 independent experiments. Data represent means ± SEM. (n = 3; 2-way ANOVA). Significant *P < 0.05, ***P < 0.001. Significant, ***P < 0.001; ns, not significant. **E.** Expression levels of proteins in WT and *jnk1/2−/−* MEFs were examined by western blot analysis in response to 1 μM GSK2606414 treatment for times indicated. Expression for P-Foxo3 (T32) (95 kDa), Foxo3 (85 kDa), Gcn2 (220 kDa), Perk (140 kDa), P-eIF2a (38 kDa), eIF2a (38 kDa), P-JNK(54 kDa), JNK (54 kDa), P-AKT (S473) (60 kDa), P-AKT (T308) (60 kDa), AKT (60 kDa) and β-Tubulin (55 kDa).

To confirm the potential reciprocal negative regulation between GCN2 and PERK, we next studied the expression of JNK, FOXO3 and PERK in the wild-type and GCN2-deficient mouse embryo fibroblasts (*gcn2*^*−/−*^ MEFs) in response to GSK2606414 and epirubicin treatment (Fig. 7; Supplementary Fig. S5). Clonogenic assays showed that GCN2 deletion caused a significant reduction in clonal viability and sensitised these MEFs to epirubicin (Supplementary Fig. S5) and PERK inhibition by GSK260641 (Fig. 7A). The Western blot analysis showed that P-FOXO3 (T32) expression level decreased in response to knockout of GCN2 (Fig. 7B; Supplementary Fig. S5). Furthermore, P-JNK and JNK expression levels were also increased in *gcn2*^−/−^ MEFs providing further evidence that GCN2 represses JNK and FOXO3 activity. In agreement, in *gcn2*^*−/−*^ MEFs total PERK and P-PERK levels were induced accompanied by FOXO3 activation, which regulates PERK at the transcriptional level (Fig. 7C). In addition, GCN2-deficient MEFs had reduced clonogenicity, confirming our earlier finding in MCF-7 cells that GCN2 has a pivotal role in promoting clonal viability (Fig. 7B).

### 3.6 JNK-deletion inactivates FOXO3 and attenuates GCN2 and PERK expression

**Fig. 8:**
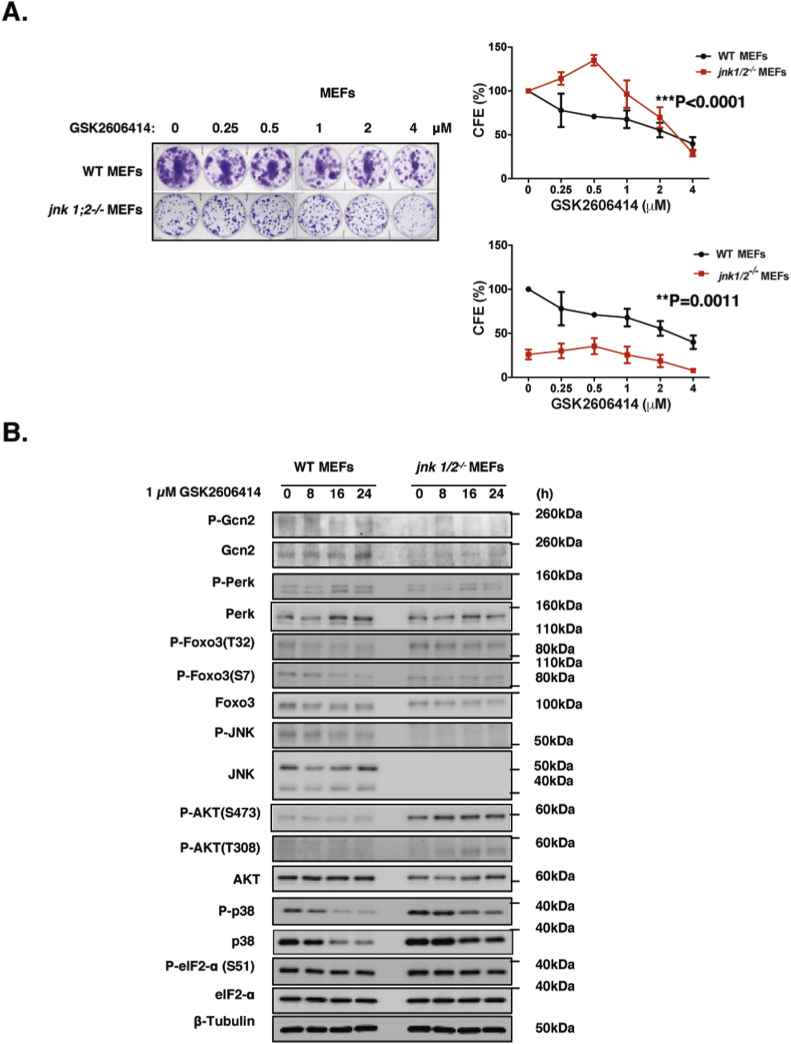
**Clonogenic assays to investigate the effects of JNK deletion and PERK inactivation in MEFs**. **A.** WT MEFs and *jnk1/2*^*−/−*^ MEFs were treated with different concentrations of GSK2606414 for 48 h. Fresh media were then added and cells maintained for 10 days before staining with crystal violet in clonogenic assays. Representative images of at least 3 independent experiments are shown. The clonogenic results of the cells were normalised to that of the WT MEFs without epirubicin treatment (Top right panel). The clonogenic results of the cells were also normalised to that of the respective WT and *jnk1/2*-deficient MEFs without GSK2606414 treatment (Bottom right panel). Data are representative of 3 independent experiments. Data represent means ± SEM. (n = 3; 2-way ANOVA). Significant *P < 0.05, ***P < 0.001. Significant, ***P < 0.001; ns, not significant. **B**. Expression levels of proteins in WT and *jnk1/2−/−* MEFs were examined by western blot analysis in response to 1 μM GSK2606414 treatment for times indicated. Expression for P-Foxo3 (T32) (95 kDa), Foxo3 (85 kDa), Gcn2 (220 kDa), Perk (140 kDa), P-eIF2a (38 kDa), eIF2a (38 kDa), P-JNK(54 kDa), JNK (54 kDa), P-AKT (S473) (60 kDa), P-AKT (T308) (60 kDa), AKT (60 kDa) and β-Tubulin (55 kDa).

Our siRNA knockdown, pharmacological inhibition and gene knockout data indicated that JNK expression and activity are induced upon GCN2 or PERK inactivation, indicating that JNK might mediate the compensatory negative feedback loop between GCN2 and PERK. Mice possess three JNK isoenzymes, JNK1, JNK2 and JNK3. Whereas JNK1 and JNK2 are ubiquitously expressed and have overlapping or redundant functions, JNK3 expression appears to be restricted to brain, heart and testis (Bode and Dong, 2007; Gupta et al., 1996). To investigate the role of JNK in the compensatory crosstalk between GCN2 and PERK, we studied the effects of JNK-deletion in MEFs derived from wild-type and *jnk1/2* double knockout mice (Sabapathy et al., 2004). Clonogenic assays showed that JNK-deletion augmented the clonogenicity and drug resistance of MEFs in response to the PERK inhibitor GSK2606414 (Fig. 7D), indicating JNK negatively regulated the clonal survival and resistance to PERK inactivation. Western blot analysis showed that the *jnk1/2*-deficient MEFs are depleted of JNK expression and that total GCN2 expression levels were induced in response to GSK2606414 treatment in WT MEFs, but were undetectable in *jnk1/2*^*−/−*^ MEFs (Fig. 7E). Similarly, P-PERK and total PERK levels were upregulated by GSK2606414 in WT MEFs but remained unchanged in *jnk1/2*^*−/−*^ MEFs. In contrast, P-FOXO3 (T32), P-AKT (T308; S473) were expressed at low levels in the WT MEFs but were induced by JNK-knockdown, indicating that JNK activates FOXO3 through repressing AKT. In addition, total FOXO3 expression levels and nuclear localization were also attenuated *jnk1/2*^*−/−*^ compared to WT MEFs (Fig. 8B and Supplementary Fig. S6). These results that the reciprocal negative feedback regulation between GCN2 and PERK is mediated through JNK.

## 4 Discussion

The endoplasmic reticulum (ER)-stress modulator PERK has been demonstrated to play a vital role in tumorigenesis and cancer development though its function as the key regulator of the UPR signalling pathway (Bi et al., 2005; Salaroglio et al., 2017; Siwecka et al., 2019). Consistently, we have also shown recently that PERK also promotes resistance to ER stress and cytotoxic drugs through the repression of FOXO3 by promoting AKT activation in breast cancer cells (Alasiri et al., 2019). In consequence, targeting PERK has emerged as a viable approach for developing novel cancer treatments (Siwecka et al., 2019). However, despite pharmaceutical inhibitors of PERK having demonstrated good anticancer activities in combination therapies, their effectiveness as a single agent is limited, suggesting the existence of possible compensatory cellular responses (Axten et al., 2012, 2013; Bi et al., 2005; Salaroglio et al., 2017). Previous studies in Drosophila have shown that GCN2 (eIF2AK4) and PERK (eIF2AK3) can cooperate to modulate FOXO activity in response to ER stress (You et al., 2018; Zhang et al., 2013), suggesting the PERK-related GCN2 can potentially compensate for the inactivation of PERK function in cancer cells. However, in contrast to our finding in human breast cancer cells (Alasiri et al., 2019), these studies in flies suggest that both GCN2 and PERK potentiate rather than repress FOXO activity in response to ER stress (You et al., 2018; Zhang et al., 2013). Nevertheless, the time-course drug treatment experiments on the parental MCF-7 and drug resistant MCF-7Epi^R^ and MCF-7Tax^R^ breast cancer cells lent support to the idea that GCN2 can potentially cooperate with PERK to repress FOXO3 activity via JNK and AKT to modulate drug response and compensate for PERK inactivation. Specifically, the cytotoxic drug time course experiments revealed that chemotherapeutics, including epirubicin and paclitaxel, caused PERK and GCN2 dephosphorylation/inactivation, which was associated with JNK activation, AKT repression, and the induction of the antiproliferative functions of FOXO3. Nevertheless, in the drug resistant MCF-7Epi^R^ and MCF-7Tax^R^ cells, PERK and GCN2 activity remained constitutively high and was not affected by drug treatments. Collectively, these findings proposed that GCN2 and PERK have overlapping functions and cooperate to integrate the upstream clonal survival and cytotoxic drug signals with downstream effectors, such as JNK, AKT and FOXO3, to modulate clonal viability and cytotoxic drug response. In agreement, our previous findings using FOXO1/3/4-deficient MEFs and FOXO3 siRNA-mediated gene silencing have demonstrated that FOXO proteins, in particular FOXO3, are essential for mediating the clonal survival as well as the cytotoxic functions of chemotherapeutic agents and PERK inhibitors (Alasiri et al., 2019; Sunters et al., 2003, 2006).

The crosstalk and reciprocal negative regulation between GCN2 and PERK and their roles in clonal viability and cytotoxic drug response were confirmed using GCN2 siRNA knockdown and the PERK inhibitor GSK2606414. GCN2 silencing using siRNA severely impaired the clonal survival of MCF-7 cells. These GCN2-depleted cells were also sensitised to epirubicin, paclitaxel and the PERK inhibitor GSK2606414, advocating that GCN2 and PERK have overlapping functions and that it is GCN2 that compensates for the functional loss of PERK in response to drug treatment. Critically, GCN2 silencing almost completely obliterated the clonal viability of the drug resistant MCF-7Epi^R^ and MCF-7Tax^R^ cells, suggesting that the drug resistant cells are heavily dependent on GCN2 for their continuous clonal survival and drug resistance. Intriguing, GCN2 silencing in MCF-7 cells did not result in a comprehensive depletion of the expression of the GCN2/PERK downstream target P-eIF2α (S51) but instead caused a reciprocal increase in PERK expression and activity, suggesting that PERK is induced to compensate for the GCN2 loss in a negative feedback loop. Similarly, blocking PERK with GSK2606414 did not affect downstream P-eIF2α (S51) signalling but instead induced the GCN2 expression and activity in MCF-7 cells, suggesting that GCN2 is also induced to alleviate the reduction in clonal survival signals due to PERK loss. GSK2606414 has been shown to be a highly potent PERK inhibitor (IC50 = 0.4 nM; [ATP] = 5 μM) by targeting PERK at the ATP-binding region, and demonstrates ≥385-fold selectivity over other related kinases, including c-Kit, Aurora B, BRK, HRI/EIF2AK1, MLK2/MAP3K10, c-MER, DDR2, PKR/EIF2AK2, and MLCK2/MYLK2 (Axten et al., 2012). However, a recent study also indicates that GSK2606414 and the related compound GSK2656157 (Axten et al., 2012, 2013) are also potent TNF-mediated RIPK1 inhibitors (Rojas-Rivera et al., 2017). As a result, the reciprocal negative feedback loop between PERK and GCN2 that mediates their compensatory cross-talk is confirmed further in the PERK and GCN2-deficient MEFs, which provide more definitive results than the data from MCF-7 cells. These gene-knockout MEFs results also indicate that chemotherapeutic agents, such as epiribicin and GSK2606414, trigger JNK and FOXO3 activation through PERK and GCN2. This cross-talk is made possible by GCN2 and PERK have overlapping functions, being able to integrate the same upstream signals with identical downstream functional effectors. Together these data also suggest that PERK and GCN2 function cooperatively to integrate the upstream clonal survival and cytotoxic agent signals with the downstream JNK, AKT and FOXO3 activity to modulate clonal viability and chemotherapeutic drug resistance. This compensatory mechanism can account for the relative insensitivity of cancer cells to PERK inhibitors alone.

Based on our collective data, we conclude that drug resistance and continuous clonal survival develops from the redundancy of PERK and GCN2 which together restrict JNK activity and thus the antiproliferative functions of FOXO3. This idea is supported by our data showing that PERK or GCN2 inactivation causes derepression of JNK expression and activity and confirmed further by our findings in JNK-deficient MEFs that both PERK and GCN2 expression and activity are attenuated. Moreover, PERK-inactivation by GSK2606414 in JNK-deficient MEFs fails to induce either GCN2 or PERK expression. The results from JNK-deficient MEFs also demonstrate that JNK has a role in promoting GCN2 expression, as GCN2 expression is constitutively attenuated in JNK-deficient MEFs compared with their wild-type counterparts. The induction of GCN2 is likely to be mediated at the post-transcriptional level as PERK inhibition in MEFs induces GCN2 overexpression at the protein but not at the transcription level.

Our present results confirm our previous findings that the drug-resistant MCF-7Epi^R^ and MCF-7Tax^R^ cells which express low PERK and high P-PERK levels are sensitive to PERK inhibition. Notably, these results also reveal that unlike the parental MCF-7 cells, the drug resistant MCF-7Epi^R^ and MCF-7Tax^R^ cells cannot be rescued by the compensatory induction of GCN2 upon PERK inhibition. Consistent with this, Western blot analysis shows that upon GSK2606414 treatment GCN2, JNK and P-JNK was induced in the MCF7 cells but not in the drug resistant MCF-7Epi^R^ and MCF-7Tax^R^ cells. This is likely to reflect the fact that GCN2 is already expressing at very high levels due to low JNK activity and cannot be induced much further by extra JNK repression in these cells.

Consistent with our results, a recent study showed that the two eIF-2α kinases, eIF2AK3 and eIF2AK4, contributed to paclitaxel response in breast cancer (Chen et al., 2019). However, while the study indicated that the eIF2AK3/eIF2AK4-P-eIF2S1-ATF4 axis contributes to paclitaxel response via modulating redox homoeostasis by transcriptionally regulating antioxidant genes, such as HMOX1, SHMT2 and SLC7A11 (Chen et al., 2019), Our present and past data suggest that the cytotoxic effects of chemotherapeutic drugs are predominantly mediated through the eIF2AK3/eIF2AK4-JNK-FOXO pathway (Alasiri et al., 2018). In support, we have shown previously that FOXO proteins mediate the cytotoxic effects of chemotherapeutic drugs and PERK inhibitor GSK2606414, which are attenuated in FOXO1/3/4-deficient fibroblasts compared with their wild-type counterparts (Alasiri et al., 2018). Furthermore, our data also indicates that FOXO3 is constitutively inactive in JNK-deficient MEFs as revealed by the FOXO3 inactivating T32-phosphorylation and the slower migrating phosphorylated FOXO3 species. Nonetheless, JNK has also been shown to be essential for ATF4 expression and activated upon ER stress, suggesting the signalling cascades involving ATF4 and FOXO3 are not mutually exclusive (Hara et al., 2017; Matsuguchi et al., 2009).

Intriguingly, our data also suggest that GCN2 is a more effective drug target than PERK in these breast cancer cells. The reason for GCN2 contributing to the higher deleteriousness compared with PERK upon its inactivation in both the drug sensitive and resistance MCF-7 cancer cells is incompletely understood. However, the fact that FOXO3 is often inactivated in cancer cells and even more so in the drug resistant cells, suggesting that the ability of FOXO3 to induce PERK in response to GCN2 inactivation is compromised in the cancer cells, and even more in the drug resistant cells. This argument is supported by the observation that the effects of GCN2 depletion on clonal survival and drug resistance is less severe in the non-cancerous MEFs compared with the drug sensitive and resistant MCF-7 breast cancer cells. This finding advocates the potential of targeting GCN2 as a therapeutic strategy for treating cancer and for overcoming drug resistance.

Collectively, our results provide strong evidence that PERK and GCN2 function cooperatively to play a pivotal role in cancer cell clonal renewal as well as cytotoxic drug resistance by suppressing FOXO3 expression via JNK. Based on our collective data, we propose that the tolerance to PERK inhibition is mediated by the reciprocal negative feedback regulation between GCN2 and PERK mediated through JNK. Specifically, GCN2 or PERK inactivation will induce JNK to promote GCN2 and PERK expression and activity. Our data also indicate that the induction of PERK expression by JNK is at least partly mediated by FOXO3 at the transcriptional level, while that of GCN2 is at the post-transcriptional level.

## CRediT authorship contribution statement

**Glowi Alasiri:** Conceptualization, Methodology, Validation, Formal analysis, Investigation, Resources, Data curation, Writing - original draft, Writing - review & editing. **Yannasittha Jiramongkol:** Conceptualization, Methodology, Validation, Formal analysis, Investigation, Data curation, Writing - original draft, Writing - review & editing. **Sasanan Trakansuebkul:** Methodology, Validation, Formal analysis, Investigation, Data curation. **Hui-Ling Ke:** Methodology, Validation, Formal analysis, Investigation, Data curation. **Zimam Mahmud:** Writing - original draft, Methodology, Formal analysis. **Kitti Intuyod:** Conceptualization, Methodology, Formal analysis, Investigation, Writing - original draft, Writing - review & editing. **Eric W.-F. Lam:** Conceptualization, Methodology, Validation, Formal analysis, Investigation, Resources, Data curation, Writing - original draft, Writing - review & editing, Supervision, Project administration.

## Declaration of competing interest

The authors declare no conflict of interest.
