# Supplementary material for: Reciprocal regulation between GCN2 (eIF2AK4) and PERK (eIF2AK3) through the JNK-FOXO3 axis to modulate cancer drug resistance and clonal survival
Source: Mol Cell Endocrinol. 2020 Sep 15;515:110932. doi: 10.1016/j.mce.2020.110932 (PMC7493713; doi:10.1016/j.mce.2020.110932)
Supplement: Multimedia component 1 [file mmc1.pptx]

## Slide 1
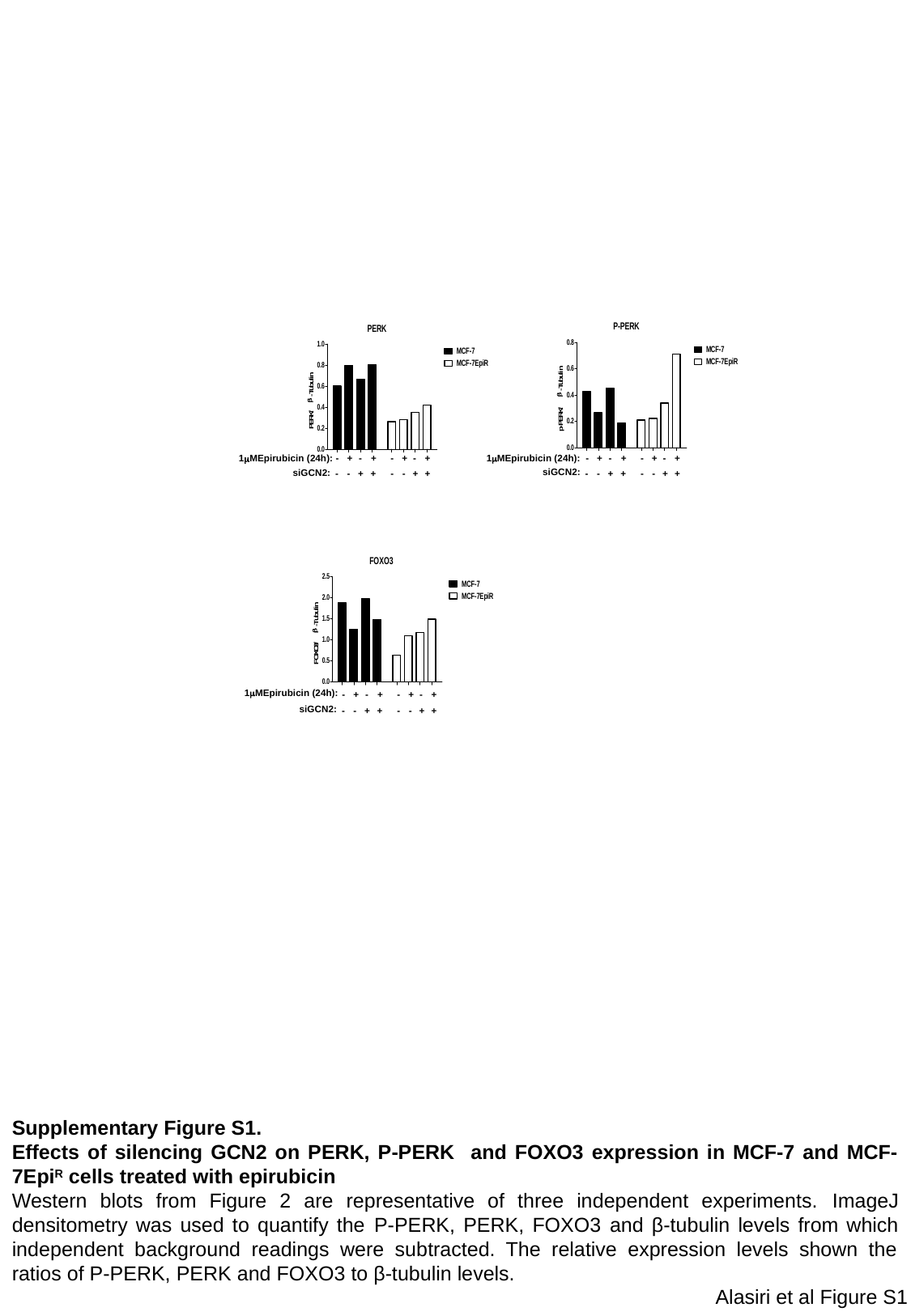

1mMEpirubicin (24h):
-
+
-
+
-
+
-
+
1mMEpirubicin (24h):
-
+
-
+
-
+
-
+
siGCN2:
siGCN2:
-
-
+
+
-
-
+
+
-
-
+
+
-
-
+
+
1mMEpirubicin (24h):
-
+
-
+
-
+
-
+
siGCN2:
-
-
+
+
-
-
+
+
Supplementary Figure S1.
Effects of silencing GCN2 on PERK, P-PERK and FOXO3 expression in MCF-7 and MCF-7EpiR cells treated with epirubicin
Western blots from Figure 2 are representative of three independent experiments. ImageJ densitometry was used to quantify the P-PERK, PERK, FOXO3 and β-tubulin levels from which independent background readings were subtracted. The relative expression levels shown the ratios of P-PERK, PERK and FOXO3 to β-tubulin levels.
Alasiri et al Figure S1
