# Supplementary material for: Reciprocal regulation between GCN2 (eIF2AK4) and PERK (eIF2AK3) through the JNK-FOXO3 axis to modulate cancer drug resistance and clonal survival
Source: Mol Cell Endocrinol. 2020 Sep 15;515:110932. doi: 10.1016/j.mce.2020.110932 (PMC7493713; doi:10.1016/j.mce.2020.110932)
Supplement: Multimedia component 3 [file mmc3.pptx]

## Slide 1
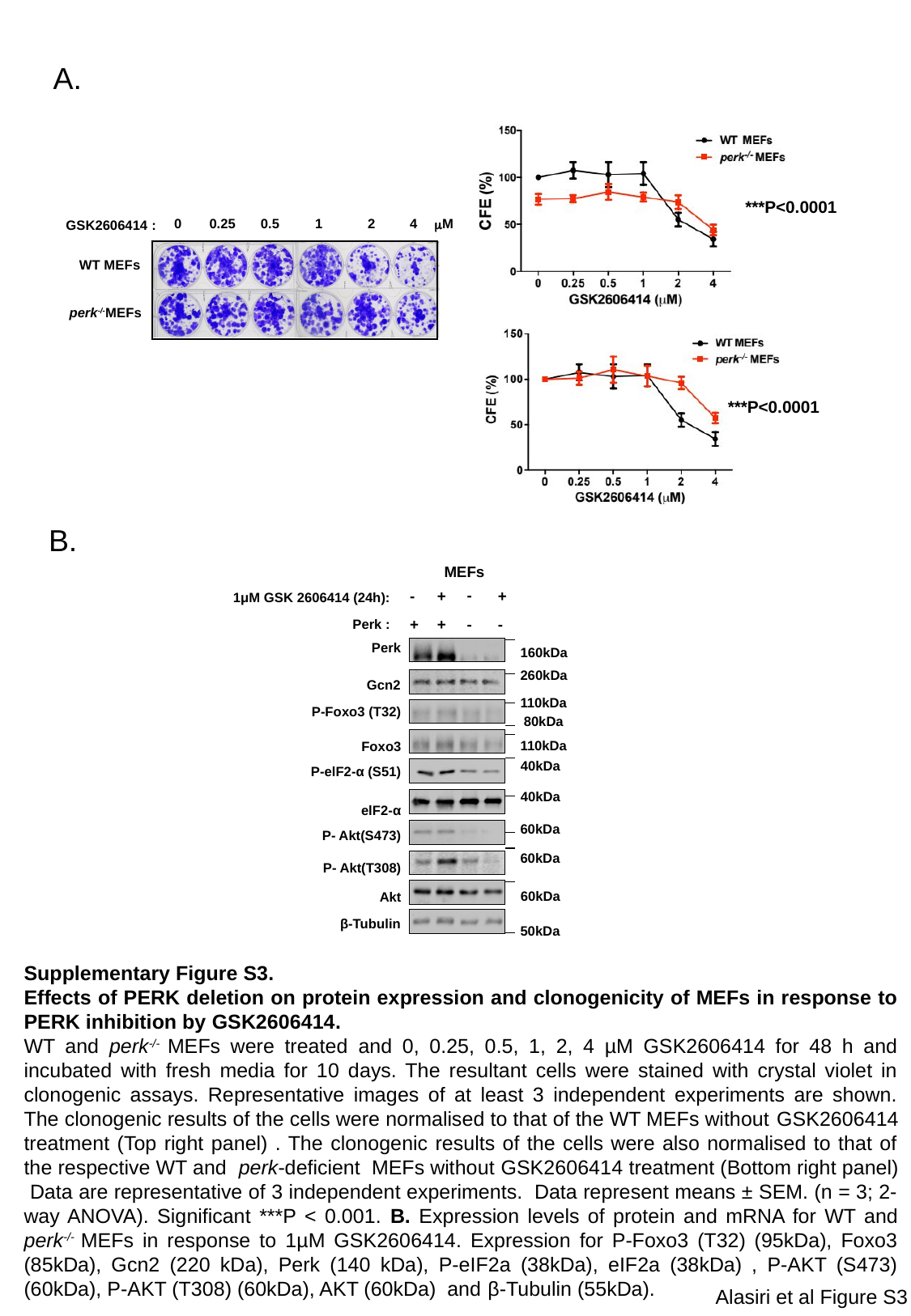

A.
***P<0.0001
0
0.25
0.5
1
2
4
mM
 GSK2606414 :
WT MEFs
perk-/-MEFs
***P<0.0001
B.
MEFs
-
-
+
+
1μM GSK 2606414 (24h):
+
+
-
-
 Perk :
Perk
160kDa
260kDa
Gcn2
110kDa
P-Foxo3 (T32)
80kDa
Foxo3
110kDa
40kDa
 P-elF2-α (S51)
40kDa
elF2-α
60kDa
P- Akt(S473)
60kDa
P- Akt(T308)
60kDa
Akt
β-Tubulin
50kDa
Supplementary Figure S3.
Effects of PERK deletion on protein expression and clonogenicity of MEFs in response to PERK inhibition by GSK2606414.
WT and perk-/- MEFs were treated and 0, 0.25, 0.5, 1, 2, 4 µM GSK2606414 for 48 h and incubated with fresh media for 10 days. The resultant cells were stained with crystal violet in clonogenic assays. Representative images of at least 3 independent experiments are shown. The clonogenic results of the cells were normalised to that of the WT MEFs without GSK2606414 treatment (Top right panel) . The clonogenic results of the cells were also normalised to that of the respective WT and perk-deficient MEFs without GSK2606414 treatment (Bottom right panel) Data are representative of 3 independent experiments. Data represent means ± SEM. (n = 3; 2-way ANOVA). Significant ***P < 0.001. B. Expression levels of protein and mRNA for WT and perk-/- MEFs in response to 1µM GSK2606414. Expression for P-Foxo3 (T32) (95kDa), Foxo3 (85kDa), Gcn2 (220 kDa), Perk (140 kDa), P-eIF2a (38kDa), eIF2a (38kDa) , P-AKT (S473) (60kDa), P-AKT (T308) (60kDa), AKT (60kDa) and β-Tubulin (55kDa).
Alasiri et al Figure S3
