# Supplementary material for: Reciprocal regulation between GCN2 (eIF2AK4) and PERK (eIF2AK3) through the JNK-FOXO3 axis to modulate cancer drug resistance and clonal survival
Source: Mol Cell Endocrinol. 2020 Sep 15;515:110932. doi: 10.1016/j.mce.2020.110932 (PMC7493713; doi:10.1016/j.mce.2020.110932)
Supplement: Multimedia component 4 [file mmc4.pptx]

## Slide 1
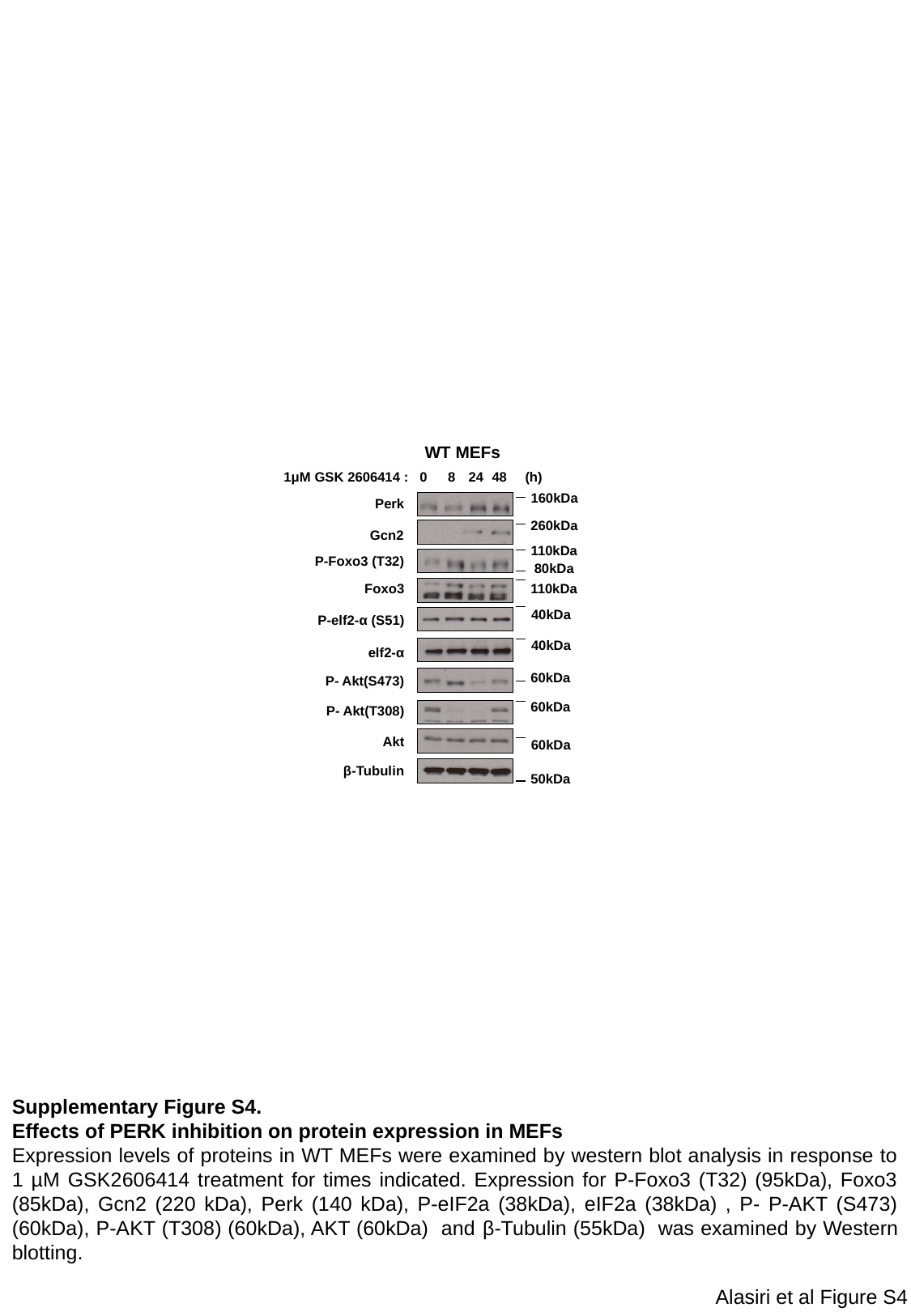

WT MEFs
1μM GSK 2606414 :
0
8
24
48
(h)
160kDa
Perk
260kDa
Gcn2
110kDa
P-Foxo3 (T32)
80kDa
Foxo3
110kDa
40kDa
 P-elf2-α (S51)
40kDa
elf2-α
60kDa
P- Akt(S473)
60kDa
P- Akt(T308)
Akt
60kDa
β-Tubulin
50kDa
Supplementary Figure S4.
Effects of PERK inhibition on protein expression in MEFs
Expression levels of proteins in WT MEFs were examined by western blot analysis in response to 1 µM GSK2606414 treatment for times indicated. Expression for P-Foxo3 (T32) (95kDa), Foxo3 (85kDa), Gcn2 (220 kDa), Perk (140 kDa), P-eIF2a (38kDa), eIF2a (38kDa) , P- P-AKT (S473) (60kDa), P-AKT (T308) (60kDa), AKT (60kDa) and β-Tubulin (55kDa) was examined by Western blotting.
Alasiri et al Figure S4
