# Supplementary material for: Reciprocal regulation between GCN2 (eIF2AK4) and PERK (eIF2AK3) through the JNK-FOXO3 axis to modulate cancer drug resistance and clonal survival
Source: Mol Cell Endocrinol. 2020 Sep 15;515:110932. doi: 10.1016/j.mce.2020.110932 (PMC7493713; doi:10.1016/j.mce.2020.110932)
Supplement: Multimedia component 5 [file mmc5.pptx]

## Slide 1
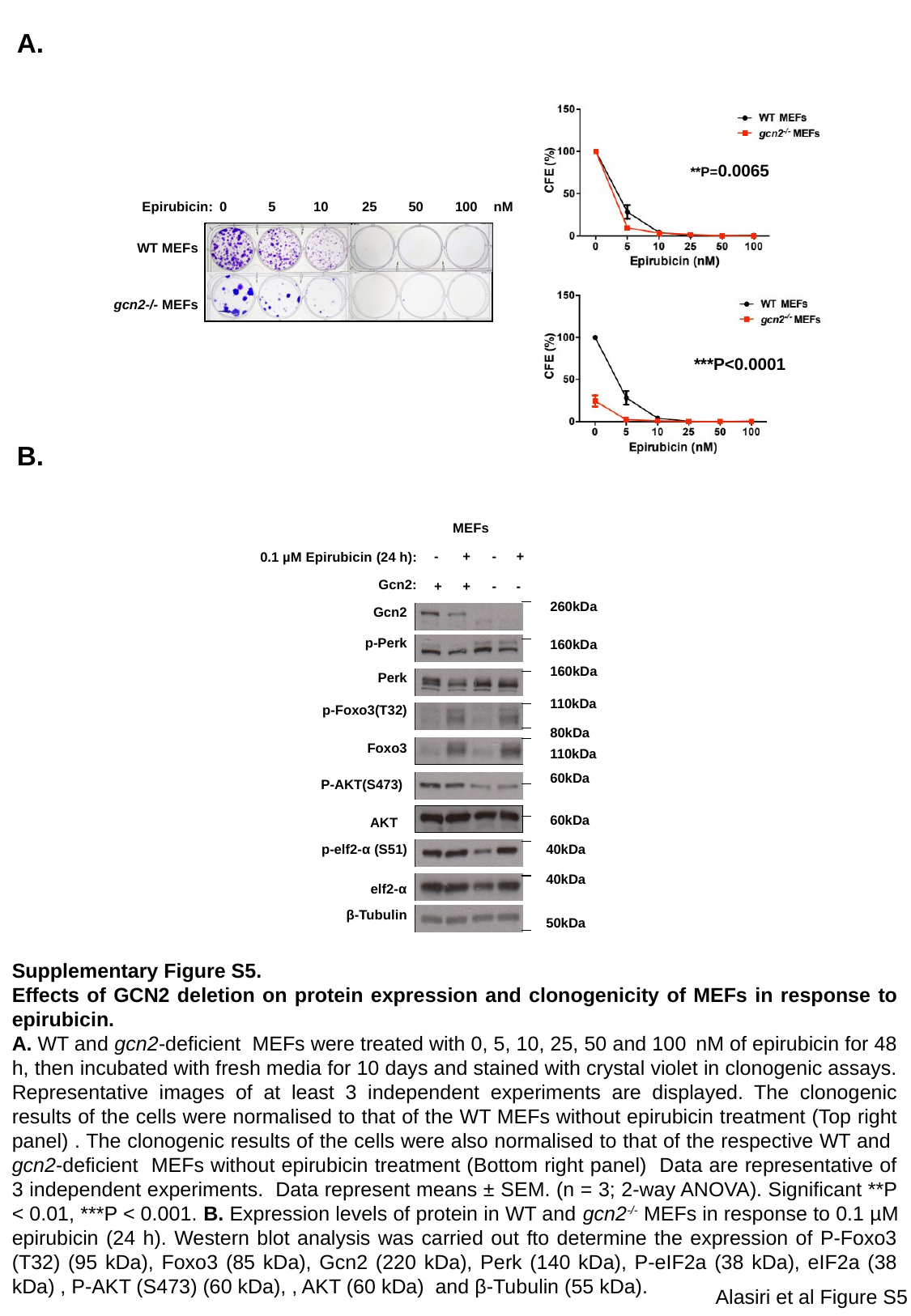

A.
**P=0.0065
Epirubicin:
0
5
10
25
50
100
nM
WT MEFs
gcn2-/- MEFs
***P<0.0001
B.
MEFs
-
+
-
+
0.1 µM Epirubicin (24 h):
 Gcn2:
+
+
-
-
260kDa
Gcn2
p-Perk
160kDa
160kDa
Perk
110kDa
p-Foxo3(T32)
80kDa
Foxo3
110kDa
60kDa
P-AKT(S473)
60kDa
AKT
40kDa
 p-elf2-α (S51)
40kDa
elf2-α
β-Tubulin
50kDa
Supplementary Figure S5.
Effects of GCN2 deletion on protein expression and clonogenicity of MEFs in response to epirubicin.
A. WT and gcn2-deficient MEFs were treated with 0, 5, 10, 25, 50 and 100  nM of epirubicin for 48 h, then incubated with fresh media for 10 days and stained with crystal violet in clonogenic assays. Representative images of at least 3 independent experiments are displayed. The clonogenic results of the cells were normalised to that of the WT MEFs without epirubicin treatment (Top right panel) . The clonogenic results of the cells were also normalised to that of the respective WT and gcn2-deficient MEFs without epirubicin treatment (Bottom right panel) Data are representative of 3 independent experiments. Data represent means ± SEM. (n = 3; 2-way ANOVA). Significant **P < 0.01, ***P < 0.001. B. Expression levels of protein in WT and gcn2-/- MEFs in response to 0.1 µM epirubicin (24 h). Western blot analysis was carried out fto determine the expression of P-Foxo3 (T32) (95 kDa), Foxo3 (85 kDa), Gcn2 (220 kDa), Perk (140 kDa), P-eIF2a (38 kDa), eIF2a (38 kDa) , P-AKT (S473) (60 kDa), , AKT (60 kDa) and β-Tubulin (55 kDa).
Alasiri et al Figure S5
