# Supplementary material for: Reciprocal regulation between GCN2 (eIF2AK4) and PERK (eIF2AK3) through the JNK-FOXO3 axis to modulate cancer drug resistance and clonal survival
Source: Mol Cell Endocrinol. 2020 Sep 15;515:110932. doi: 10.1016/j.mce.2020.110932 (PMC7493713; doi:10.1016/j.mce.2020.110932)
Supplement: Multimedia component 6 [file mmc6.pptx]

## Slide 1
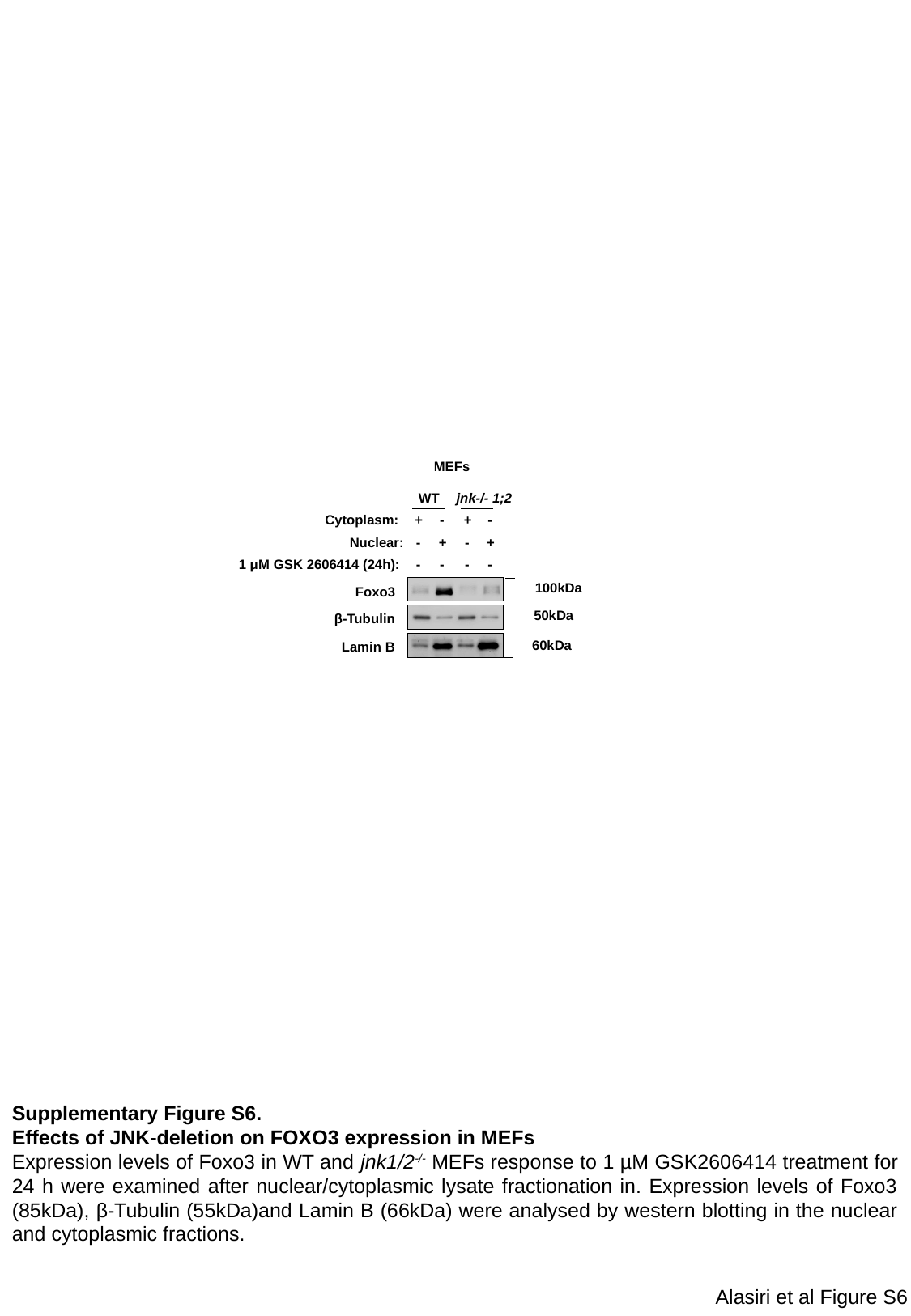

MEFs
WT
jnk-/- 1;2
Cytoplasm:
+
-
+
-
Nuclear:
-
+
-
+
1 μM GSK 2606414 (24h):
-
-
-
-
100kDa
Foxo3
50kDa
β-Tubulin
60kDa
Lamin B
Supplementary Figure S6.
Effects of JNK-deletion on FOXO3 expression in MEFs
Expression levels of Foxo3 in WT and jnk1/2-/- MEFs response to 1 µM GSK2606414 treatment for 24 h were examined after nuclear/cytoplasmic lysate fractionation in. Expression levels of Foxo3 (85kDa), β-Tubulin (55kDa)and Lamin B (66kDa) were analysed by western blotting in the nuclear and cytoplasmic fractions.
Alasiri et al Figure S6
